# RNAi screening of subtracted transcriptomes reveals tumor suppression by taurine-activated GABA_A_ receptors involved in volume regulation

**DOI:** 10.1371/journal.pone.0196979

**Published:** 2018-05-22

**Authors:** Camiel L. C. Wielders, Pim van Nierop, Tinke L. Vormer, Floris Foijer, Joanne Verheij, Johannes C. Lodder, Jesper B. Andersen, Huibert D. Mansvelder, Hein te Riele

**Affiliations:** 1 Netherlands Cancer Institute, Division of Tumor Biology and Immunology, Amsterdam, The Netherlands; 2 VU University, Center for Neurogenomics and Cognitive Research, Amsterdam, The Netherlands; 3 University Medical Centre Groningen, ERIBA, Groningen, The Netherlands; 4 Academic Medical Center, Division of Pathology, Amsterdam, The Netherlands; 5 University of Copenhagen, Biotech Research and Innovation Centre, Copenhagen, Denmark; University of South Alabama Mitchell Cancer Institute, UNITED STATES

## Abstract

To identify coding and non-coding suppressor genes of anchorage-independent proliferation by efficient loss-of-function screening, we have developed a method for enzymatic production of low complexity shRNA libraries from subtracted transcriptomes. We produced and screened two LEGO (Low-complexity by Enrichment for Genes shut Off) shRNA libraries that were enriched for shRNA vectors targeting coding and non-coding polyadenylated transcripts that were reduced in transformed Mouse Embryonic Fibroblasts (MEFs). The LEGO shRNA libraries included ~25 shRNA vectors per transcript which limited off-target artifacts. Our method identified 79 coding and non-coding suppressor transcripts. We found that taurine-responsive GABA_A_ receptor subunits, including GABRA5 and GABRB3, were induced during the arrest of non-transformed anchor-deprived MEFs and prevented anchorless proliferation. We show that taurine activates chloride currents through GABA_A_ receptors on MEFs, causing seclusion of cell volume in large membrane protrusions. Volume seclusion from cells by taurine correlated with reduced proliferation and, conversely, suppression of this pathway allowed anchorage-independent proliferation. In human cholangiocarcinomas, we found that several proteins involved in taurine signaling via GABA_A_ receptors were repressed. Low GABRA5 expression typified hyperproliferative tumors, and loss of taurine signaling correlated with reduced patient survival, suggesting this tumor suppressive mechanism operates *in vivo*.

## Introduction

Anchorage-independent proliferation, a hallmark of transformation, involves deregulation of oncogenes and tumor suppressor genes [1]. To identify coding and non-coding suppressor genes that prevent anchorless growth of primary mouse embryonic fibroblasts (MEFs), we have developed a method for efficient loss-of-function screening of enzymatically-produced low complexity shRNA libraries.

We previously showed that MEFs lacking the G1/S checkpoint proteins pRb and p107 (*Rb*^*-/-*^*107*^*-/-*^ or DKO MEFs) were immortal and refractory to senescence induced by expression of oncogenic RAS^V12^ (DKO RAS^V12^ MEFs) [2,3]. Yet, DKO RAS^V12^ MEFs were not completely transformed: they were incapable to proliferate upon removal of anchoring and did not form tumors when injected into nude mice [4]. In addition to loss of the G1/S checkpoint and RAS activation, full transformation of the cells required attenuation of the p53/p21 pathway, which could be achieved by knockdown of the transcription factor p53 (DKO RAS^V12^ p53kd MEFs), ectopic expression of the transcriptional repressor TBX2 (DKO RAS^V12^ TBX2 MEFs), or by knockdown of p21 [5,6].

To identify additional tumour suppressor genes that are repressed when DKO RAS^V12^ cells fully transform, we enzymatically produced low-complexity shRNA libraries that were subsequently used for loss-of-function screening. We reasoned that, rather than using genome-wide libraries, shRNA libraries to screen for suppressor genes should preferentially target the coding and non-coding transcripts that operate in the non-transformed cells, but whose expression is reduced in the transformed cells, as especially these transcripts are suspected to prevent transformation. To achieve this, we first optimized PCR-based Suppression Subtractive Hybridization (SSH) [7] of cDNAs synthesized from non-transformed and transformed counterparts to enrich for the polyadenylated RNAs that are repressed in transformed cells (putative tumour suppressor genes), to remove transcripts that are induced in transformed cells (putative oncogenes), and to normalize remaining transcripts present in the transcriptome (*i*.*e*., abundant and scarce transcripts are equalized). Subsequently, the selected transcripts are enzymatically processed [8,9] into a LEGO (Low-complexity by Enrichment for Genes shut Off) library of pRETRO Super vectors that can suppress the transcripts they were derived from by RNA interference. Finally, LEGO libraries are screened for transforming shRNAs to identify tumor suppressor genes.

We used our method to produce two LEGO shRNA libraries enriched for vectors targeting the polyadenylated transcripts that operate in arrested non-transformed DKO RAS^V12^ MEFs, but are reduced in transformed DKO RAS^V12^ p53kd MEFs or DKO RAS^V12^ TBX2 MEFs. Screening these LEGO libraries for vectors conferring anchorage-independent proliferation identified familiar p53/p21 pathway components. In addition, our method revealed a novel anti-proliferative mechanism in MEFs, which operates by signaling through taurine-responsive GABA_A_ receptors. This causes seclusion of cellular volume in large bleb-like protrusions, thus hindering volume growth required for sustained proliferation [10,11,12]. We found indications that taurine signaling via GABA_A_ receptors is a tumor suppressive mechanism in human cholangiocarcinoma.

## Results

### Enzymatic production of LEGO shRNA libraries from subtracted transciptomes

To produce LEGO libraries, we designed a set of oligonucleotide adapters A, B, and C (Figures A, B in S1 Fig) that allow (1) highly optimized PCR-based Suppression Subtractive Hybridization (SSH) of two transcriptomes [7] to selectively amplify cDNA sequences from repressed polyadenylated transcripts (see for schematic design Figures C, D in S1 Fig), and (2) subsequent enzymatic processing of selected cDNA sequences into a library of pRETRO Super shRNA vectors for screening (Figure I in S1 Fig). Protocols for SSH and enzymatic shRNA synthesis were tested and optimized using two isogenic cell lines of which one expressed a hygromycin resistance gene (*hyg*^*+*^) and was used as tester, whereas the other (*hyg*^*-*^*)* served as driver. The abundance of *hyg* transcripts after SSH was measured to monitor optimization (Figures E-H in S1 Fig). Because SSH employs *Alu*I fragments, two shRNA vectors can be produced from each *Alu*I site (AGCT) present in a selected sequence. Because of the abundance of *Alu*I sites, LEGO libraries generally contain many vectors targeting the same transcript, a majority causing between 40% and 80% knockdown as exemplified by vectors targeting *hyg* (Figure J in S1 Fig).

### Production of LEGO libraries enriched for suppressors of anchorless growth

**Fig 1:**
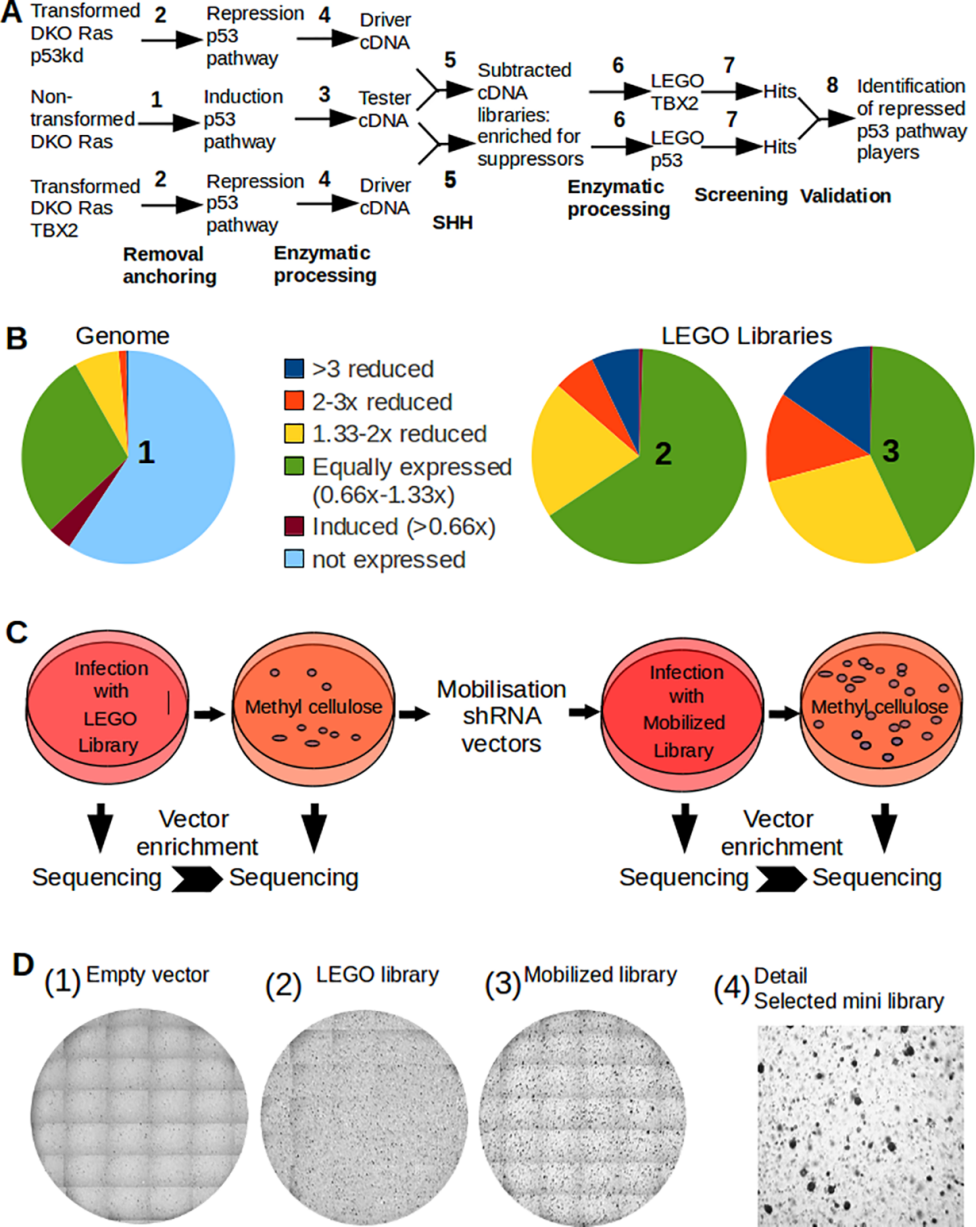
Production and screening of LEGO libraries targeting suppressors of anchorless growth. **(A)** Strategy to screen subtracted transcriptomes for suppressors of anchorless proliferation. **1.** Arrested anchor-deprived non-transformed DKO Ras^V12^ cells express suppressors. **2.** Proliferating anchorless DKO Ras^V12^ p53kd or DKO Ras^V12^ TBX2 cells repress p53 pathway players. **3.** SSH adaptors are ligated to *Alu*I-digested cDNA to yield tester cDNA. **4.** *Alu*I digested cDNA serves as driver cDNA. **5.** SSH yields two cDNA libraries enriched for sequences induced in non-transformed cells, but not in the transformed cells. **6.** SSH Adapters allow enzymatic processing of shRNA libraries LEGO-TBX2 and LEGO-p53kd. **7.** Screening LEGO-TBX2 and LEGO-p53kd for suppressors of anchorless proliferation. **8.** Validation by comparison of hits from LEGO-TBX2 and LEGO-p53kd. (**B)** Composition of the LEGO libraries. **1.** Pie diagram depicting genome-wide differential expression levels in anchorless arrested DKO RAS^V12^ MEFs and proliferating DKO RAS^V12^ TBX2 cells (DKO RAS^V12^ p53kd cells behave similar; S1 Table). The share of transcripts repressed to varying extends in transformed cells is shown. Note the abundance of non-expressed genes. **2.** Composition of the LEGO-TBX2 library as determined by deep sequencing with respect to differential expression levels of target transcripts. **3.** Composition of the LEGO-p53kd library as determined by deep sequencing with respect to differential expression levels of target sequences. **(C)** Schematic representation of the screening procedure. DKO RAS^V12^ MEFs infected with LEGO libraries were seeded in methylcellulose and vectors stimulating anchor-independent proliferation selected. Colonies were isolated from methylcellulose by centrifugation, shRNA inserts amplified by PCR, and a mobilized library was produced to perform a second round of screening. The transforming vectors were identified after deep sequencing by determining copy numbers in samples taken before and after selection. (**D)** Colonies formed by 10^6^ cells seeded in methylcellulose during a screening experiment. **1.** Mock infected DKO RAS^V12^ MEFs did not form colonies. **2.** Cells infected with a LEGO library generated small colonies during the first round of screening. **3.** Retesting of the mobilized shRNA inserts resulted in numerous small colonies. **4.** Detail of 3.

To identify novel tumor suppressor pathways, we produced and screened two LEGO (Low-complexity by Enrichment for Genes shut Off) shRNA libraries that focus on putative suppressors of anchorage-independent growth. These libraries were enriched for shRNA vectors that target genes operating in non-transformed MEFs, but being repressed in either DKO RAS^V12^ TBX2 or DKO RAS^V12^ p53kd MEFs that were transformed by attenuated p53 function (Fig 1A).

First, cDNA was synthesized from non-transformed, anchor-deprived DKO RAS^V12^ MEFs that became arrested by induction of suppressor transcripts such as p21 (tester cDNA). In addition, cDNA was produced from both transformed counterparts that formed colonies upon loss of anchoring, because suppressor transcripts were no longer induced (driver cDNAs). After SSH, both subtracted cDNA libraries were processed into shRNA vectors yielding the LEGO-p53kd and LEGO-TBX2 libraries (Fig 1A). We determined the composition of the LEGO libraries by deep sequencing, and compared it to a hypothetical shRNA library targeting the complete genome (Fig 1B). Genome-wide expression levels of protein coding genes in transformed and non-transformed cells were determined by micro array analyses (not shown). We found that 60% of the (protein coding) genes in the genome was not expressed in arrested DKO RAS^V12^ MEFs, and therefore not worthwhile screening (Fig 1B; S1 Table). In contrast to a genome-wide library, LEGO libraries only target expressed genes. Moreover, genes that were repressed over 3-, 2- or 1.33-fold in transformed MEFs represent 0.5%, 1.4%, and 7% of the genes in the genome, respectively. In contrast, in the LEGO libraries, repressed genes were represented at 11.5%, 10%, and 24.5%, respectively, thus achieving up to 21-fold enrichment (Fig 1B; S1 Table).

### Identification of suppressors of anchorless growth

Subsequently, transforming shRNA vectors in the LEGO-TBX2 and LEGO-p53kd libraries that promoted anchorage-independent growth were identified according to the scheme depicted in Fig 1C. First, non-transformed DKO RAS^V12^ MEFs were transduced with the LEGO libraries and seeded into methylcellulose. Infection with the LEGO libraries promoted the formation of small colonies (Fig 1D). These colonies were isolated by diluting the methylcellulose and separating the colonies from single (non-transformed) cells by centrifugation. Integrated shRNA sequences present in the pool of colonies were amplified by PCR and reinserted into pRETRO Super backbones, followed by a second round of selection. Numerous small colonies now formed, showing that transforming vectors inducing anchorage-independent growth had been selected from the LEGO library (Fig 1D). For suppressor gene profiling, we first determined the short hairpin sequences of the transforming LEGO shRNA vectors present in the anchorless proliferating colonies by deep sequencing, and subsequently used BLAST to identify their target transcripts. To minimize off-target effects, we took advantage of the fact that the two LEGO libraries contained multiple shRNAs targeting the same transcript. We selected transcripts that were targeted by 3 or more different transforming shRNA vectors selected independently from both the LEGO-TBX2 and LEGO-p53kd library. These transcripts were ranked according to the total number of transforming vectors identified per transcript and the share of *Alu*I sites in the transcript that resulted in a transforming shRNA vector. S2 Table lists the 79 top-ranking suppressors of anchorless proliferation identified in our screens; a selection of genes further studied is shown in Table 1. Microarray analysis indicated that, among the 79 putative suppressor transcripts, 32 were induced (>1.66 fold) in non-transformed MEFs upon loss of anchoring and 35 were repressed (>1.66 fold) in either anchorless growing DKO RAS^V12^ TBX2 cells (n = 28), DKO RAS^V12^ p53kd cells (n = 33), or both genotypes (n = 26).

**Table 1:** Selected suppressors of anchorless proliferation. A selection of genes involved in suppression of anchorless proliferation is listed. The complete list is found in S2 Table. The absolute number of transforming vectors identified per suppressor transcript by screening both LEGO-TBX2 and LEGO-p53 is shown as well as the percentage these vectors constitute of the maximum number of vectors that could enzymatically be produced from each *Alu*I site (see also Figure I in S1 Fig). Suppressor transcripts were ranked according to the score obtained by multiplying the absolute number of identified transforming vectors and the percentage of transforming shRNA vectors produced per transcript. In addition, its induction in DKO RAS^V12^ MEFs after loss of anchoring, and its repression in anchorless DKO RAS^V12^ TBX2 or DKO RAS^V12^ p53kd cells compared to anchorless DKO RAS^V12^ cells are shown. Genes reduced >1.66 fold in anchorless DKO RAS^V12^ TBX2 or DKO RAS^V12^ p53kd cells are shown in bold, genes induced >1.66 fold in DKO RAS^V12^ after loss of anchoring are shown in italics.

Gene	Ranking	Absolute no. of transforming vectors	Percentage of transforming vectors	Repression by TBX2	Repression by p53	Induction by loss of anchor
MK3	1092	14	78	**1.8**	**2.1**	*2*.*0*
p21	477	9	53	**2.2**	**2.2**	*2*.*6*
Tug1	352	16	22	1.4	**1.7**	1.4
Actr2	203	7	29	0.8	1.0	1.0
Ado	184	8	23	1.3	0.8	1.1
Lrrc8a	176	4	44	0.8	0.7	0.9
Gabarap	176	4	44	**1.8**	1.3	*1*.*9*
Mir34a	176	8	22	**2.2**	**2.1**	*2*.*1*
GabrB3	156	6	26	**2.1**	**3.5**	*4*.*3*
Arpc1b	145	5	29	1.1	0.9	1.0
Moesin	132	6	22	0.8	0.9	0.6

We first validated our hit list by designing and ordering novel shRNA vectors targeting a selection of 17 coding and non-coding suppressor transcripts (Figures A-C in S2 Fig). Knockdown of each of these 17 transcripts in DKO RAS^V12^ cells yielded colonies in methylcellulose, confirming their growth suppressive activity in anchorless cells and showing that our screening approach and the subsequent selection criteria used indeed minimized off-target effects (Figures C-E in S2 Fig).

Seventeen of the putative suppressor transcripts were non-coding RNAs, including the reported p53 targets TUG1 and miR-34a [13,14], which were identified by 16 and 8 different shRNA vectors, respectively (Table 1). We confirmed the growth suppressive role of miR-34a: knockdown of the miR-34a precursor stimulated anchorless proliferation while, in contrast, overexpression of miR-34a reverted TBX2-induced transformation (Figure D in S2 Fig).

**Fig 2:**
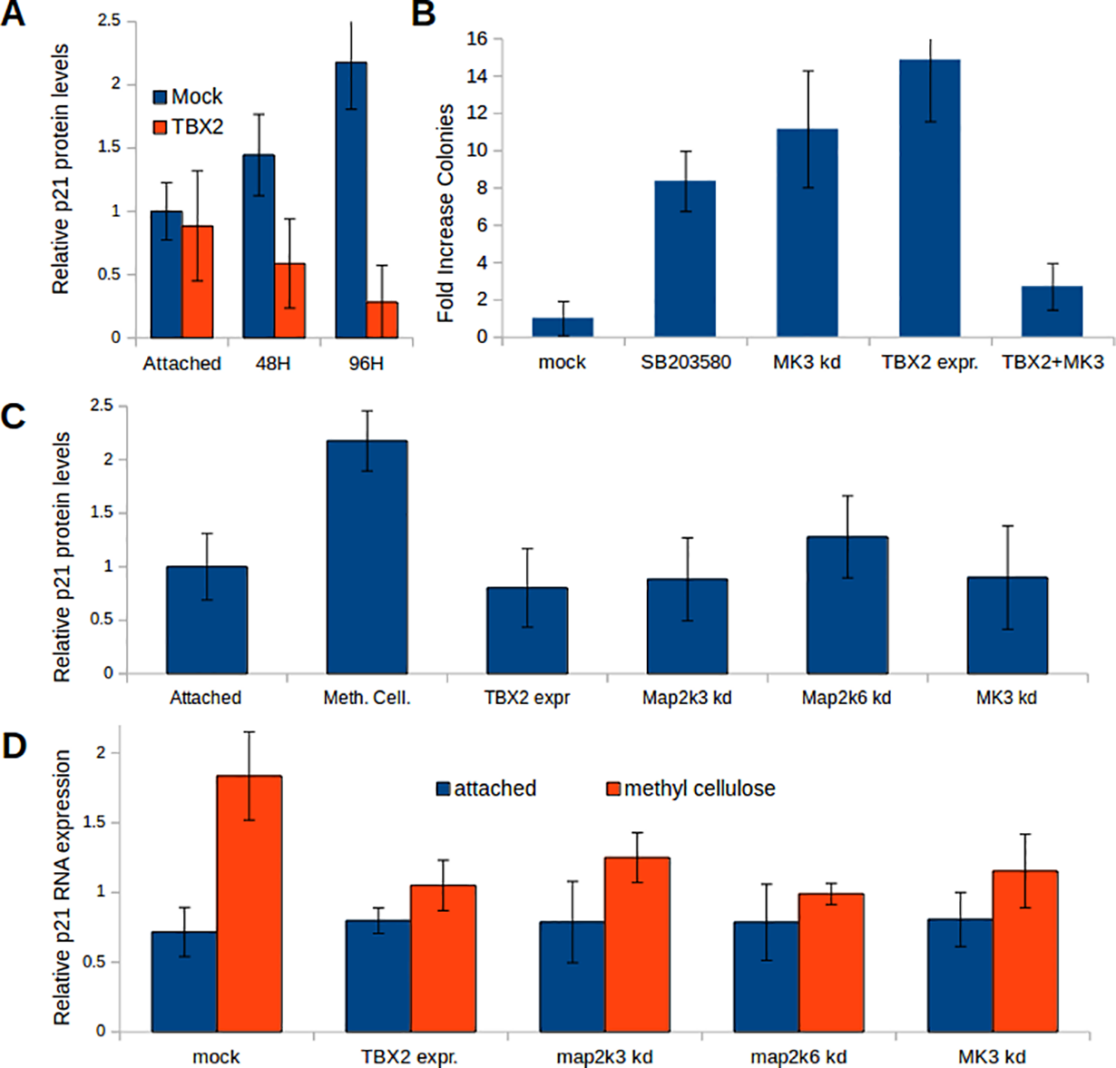
p38 stress signaling suppresses anchorless proliferation. **(A)** Quantification of p21 protein induction measured at different time points in three independent experiments showing that induction of p21 protein in DKO RAS^V12^ MEFs (blue) after seeding in methylcellulose was prevented by TBX2 (red). For Western Blot images see S3 Fig. **(B)** Colony-forming assays in methylcellulose implicate p38-mediated stress signaling in suppression of anchorless proliferation. Cells treated with the p38 inhibitor SB203580 or subjected to MK3 knockdown formed colonies in methylcellulose. In contrast, ectopic expression of MK3 reduced colony formation of TBX2-transformed cells (TBX2+MK3). Colonies were counted 3 weeks after seeding 5x10^4^ cells per well. **(C)** Quantification of four independent western blots showing p21 induction 4 days after removal of anchoring. Expression of TBX2 and knockdown of p38 pathway players prevented the induction of p21. **(D)** p21 RNA levels measured in four independent experiments by QPCR in attached cells (blue) or 2.5 days after removal of anchoring (red). Knockdown of p38 pathway players reduced p21 RNA to the same level as TBX2 expression.

Nine transforming LEGO vectors targeting the previously validated suppressor gene p21 [6] were selected during screening, again illustrating the robustness of our method (Table 1). We confirmed that TBX2 expression, like p53 knockdown, prevented the two-fold induction of p21 protein that caused arrest in non-transformed cells upon loss of anchoring (Fig 2A; for Western Blot images see S3 Fig). Ingenuity analysis of the repressed suppressor genes revealed an anti-proliferative network including p53, p21 and the stress kinase p38 that was attenuated during transformation (Figure F in S2 Fig). MK3, an effector kinase of the stress-activated kinase p38 was the highest-ranking suppressor of anchorless growth repressed in both transformed genotypes and identified by 14 knockdown vectors (Table 1). Both, the chemical p38 inhibitor SB203580 and knockdown of MK3 stimulated anchorless colony formation (Fig 2B, Figure E in S2 Fig). Moreover, we found that viral over-expression of MK3 strongly counteracted the transforming capacity of TBX2 (Fig 2B, Figure E in S2 Fig). shRNA-mediated knockdown of MK3 or the p38 activating kinases Map2k6 and Map2k3 (Figure E in S2 Fig) prevented p21 RNA and protein induction upon removal of anchoring, similar to TBX2 expression (Fig 2C and 2D).

Together, our screening method identified a familiar tumor suppressor pathway and revealed that p21-mediated suppression of anchorless proliferation of non-transformed DKO RAS^V12^ MEFs involves a p38-mediated stress response via MK3, which is attenuated in the transformed genotypes.

### GABA_A_ receptors on MEFs and taurine suppress anchorless proliferation

**Fig 3:**
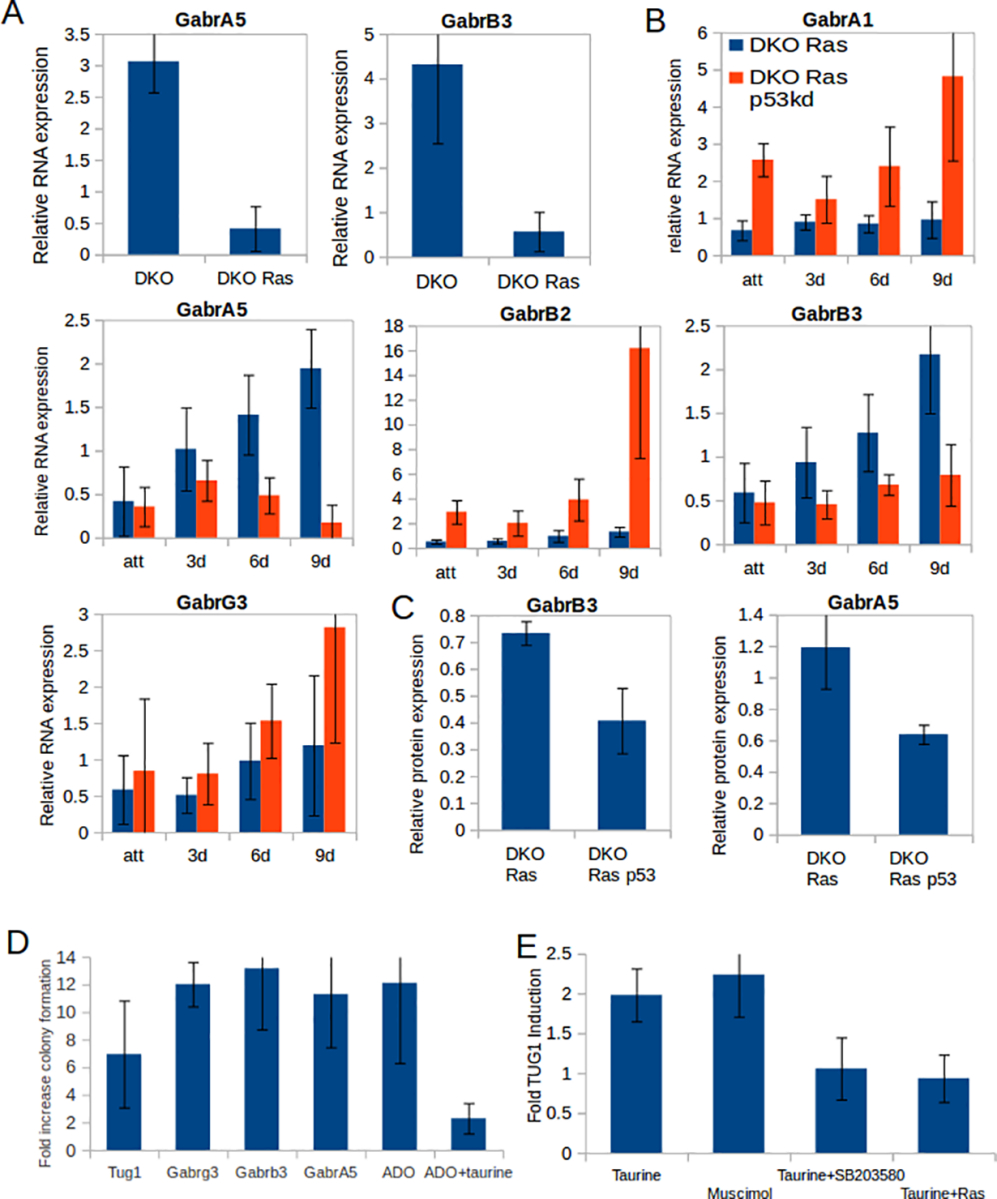
Taurine and GABA receptors are involved in suppression of anchorless proliferation. **(A)** RAS^V12^ reduced expression of GabrA5 and GabrB3 in attached DKO cells. (**B**) RNA expression of GABA receptor subunits A1, A5, B2, B3 and G3 in DKO RAS^V12^ (blue) and in DKO RAS^V12^ p53kd cells (red) after 0 (att), 3, 6 and 9 days of anchorless culturing. **(C)** Quantification of western blots (for Western Blot images see S3 Fig) showing reduction of GabrA5 and GabrB3 protein levels in anchor-deprived DKO RAS^V12^ cells upon knockdown of p53. **(D)** DKO RAS^V12^ cells in which the GABA receptor subunits A5, B3, or G3, ADO, or TUG1 were knocked down were seeded in methylcellulose, and the number of colonies formed after 2 weeks was compared to mock infected cells. The transforming effect of ADO knockdown was reverted by addition of taurine (for colony images see Figure G in S2 Fig). **(E)** Expression of TUG1 relative to HPRT as measured by quantitative PCR. The induction of TUG1 in attached DKO cells seen after 6 days of treatment with taurine (150 μM) or muscimol (100 μM) was prevented by SB20358 (10 μM) or Ras^V12^ expression.

In addition, our method revealed a novel suppressor mechanism of anchorless proliferation. The GABA_A_ receptor subunit GABRB3 and the taurine-responsive non-coding RNA TUG1 [15] were identified as suppressors that were repressed in transformed RAS^V12^ p53kd MEFs 3- and 2-fold, respectively (Table 1). We also identified three other proteins as suppressors in our screen: 2-aminoethanethiol dioxygenase (ADO), which is involved in the production of the suspected GABA_A_ receptor agonist taurine from Coenzyme A (CoA) [16], LRRC8a, which is involved in taurine excretion during volume reduction [17,18], and the GABA_A_ receptor associated protein GABARAP (Table 1). Therefore, we investigated whether MEFs harbor functional taurine-activated GABA_A_ receptors that suppress anchorless proliferation. GABA_A_ receptors are pentameric ligand-gated chloride channels that typically consist of two alpha-type, two beta-type, and single gamma-type subunits. RNA sequencing revealed that anchorless DKO RAS^V12^ MEFs sparsely expressed the GABA_A_ subunits GABRA1, GABRA5, GABRB2, GABRB3, and GABRG3 (S4 Fig). Quantification of subunit expression by PCR showed that the composition of GABA_A_ receptors on MEFs was modulated by RAS^V12^ expression, loss of anchoring and p53 function (Fig 3). Expression of RAS^V12^ in attached DKO MEFs diminished GABRA5 and GABRB3 expression (Fig 3A). During the proliferative arrest of anchor-deprived non-transformed DKO RAS^V12^ cells, GABRA5 and GABRB3 were induced approximately four-fold compared to adherent cells (Fig 3B). In contrast, the transformed DKO RAS^V12^ p53kd MEFs did not induce GABRA5 and GABRB3 RNA and protein upon loss of anchoring (Fig 3B and 3C; for Western Blot images see S3 Fig), but expressed the GABRA1 and GABRB2 subunits.

Next, testing of additionally designed shRNA vectors confirmed that the individual knockdown of three subunits of the GABA_A_ receptor complex, GABRA5, GABRB3 and GABRG3, stimulated anchorless proliferation of DKO RAS^V12^ cells, as did knockdown of ADO, and, to a lesser extent, TUG1 (Fig 3D; S2 Fig). Moreover, supplementation of ADO knockdown cells with taurine again imposed growth arrest in methylcellulose (Fig 3D; for colony images see Figure G in S2 Fig). Furthermore, prolonged exposure (6 days) to taurine (150 μM) or muscimol (100 μM), a specific agonist of GABA_A_ receptors, induced TUG1 expression in adherent DKO MEFs similarly (Fig 3E). Like p21 expression, induction of TUG1 by taurine depended on p38-mediated stress signaling, and it was balanced by RAS activity: induction was prevented by the p38 inhibitor SB203580 and by expression of RAS^V12^ (Fig 3E).

### Taurine-activated GABA_A_ receptors cause volume seclusion in membrane protrusions associated with reduced proliferation

**Fig 4:**
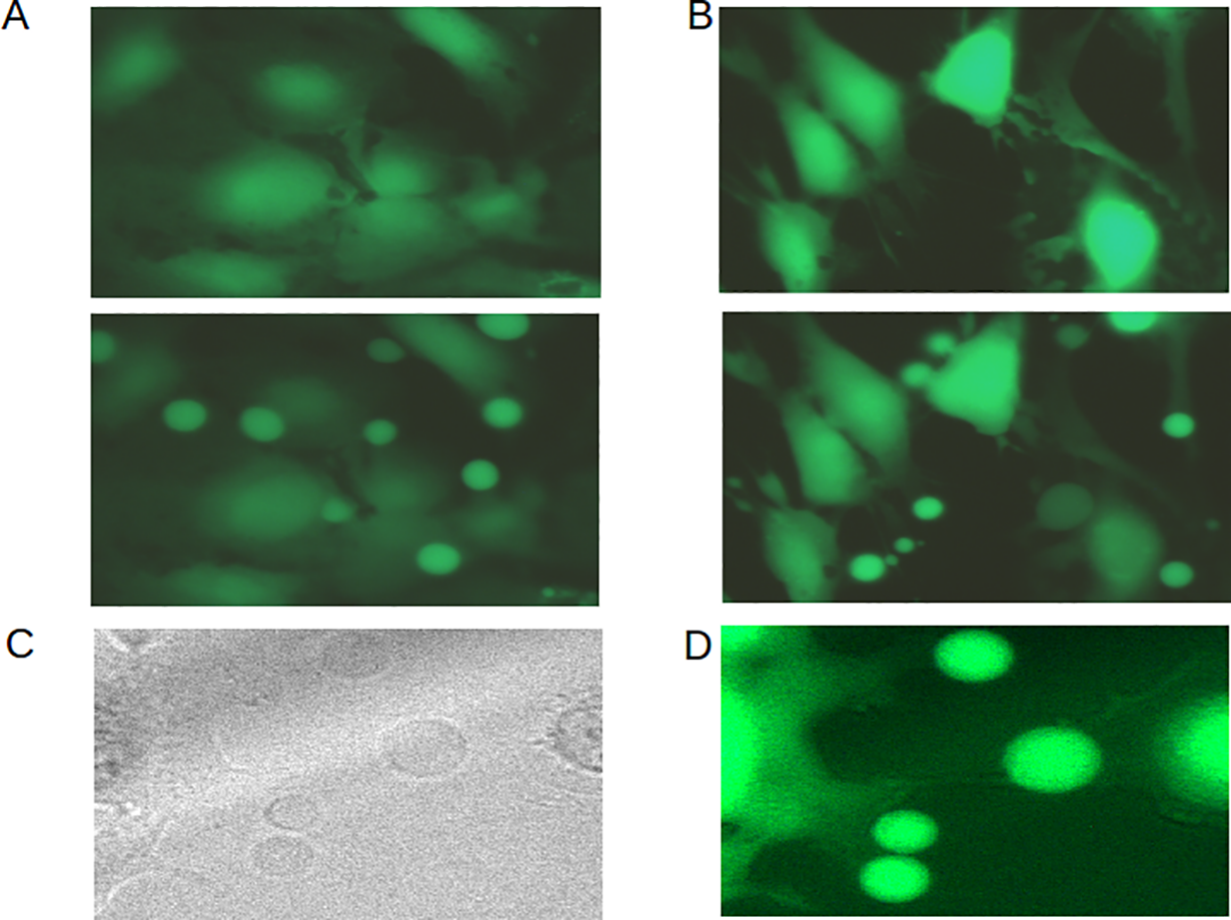
Regulation of GABA receptors correlates with protrusion formation and proliferation. **(A)** Cells labeled with green cytosolic fluorescent dye (calcein-AM) were followed by time-lapse photography (S1 Video). Fluorescence micrographs of attached DKO MEFs before (upper panel) and 5 min after (lower panel) exposure to hyposmotic shock show formation of voluminous protrusions, which sequester the fluorescent dye. **(B)** Fluorescence micrographs of attached DKO MEFs before (upper panel) and 45 min after (lower panel) exposure to taurine (500 μM) show a similar response (S2 Video). **(C, D)** Detail of protrusions that appear to be formed at pseudopods (**C** is light-microscopic image of **D**).

**Fig 5:**
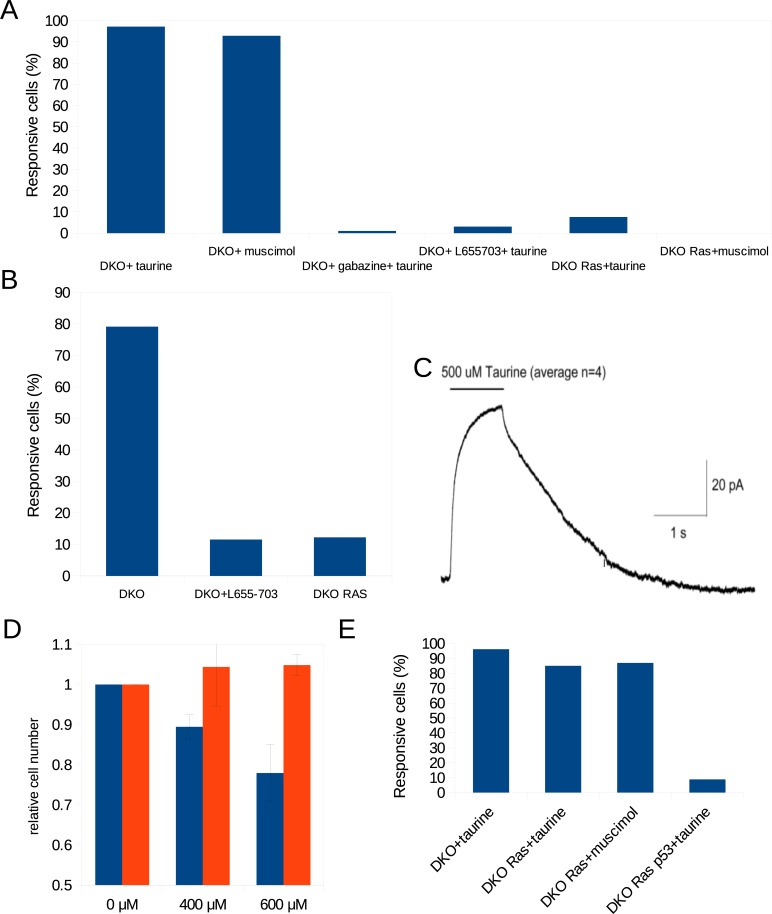
Regulation of GABA receptors correlates with protrusion formation and proliferation. **(A)** Quantification of protrusion formation in adherent DKO MEFs treated with taurine (500 μM; S2 Video), muscimol (100 μM; S3 Video), taurine (500 μM)+gabazine (10 μM; general inhibitor of GABA_A_ receptors; S4 Video), taurine (500 μM)+L655703 (100 nM; specific antagonist of GABRA5; S5 Video), as well as DKO RAS^V12^ MEFs treated with taurine (S6 Video) or muscimol. The percentage of cells that respond within 1 h is shown. **(B)** The GABRA5 antagonist L655703 (100 nM; S6 Video) and Ras^V12^ expression suppressed the formation of protrusions in attached DKO cells subjected to hyposmotic shock. The percentage of cells with protrusions after 10 min is indicated. **(C)** Current across the fibroblast membrane in response to 1 s pulse application of taurine (500 μM) measured by patch clamp. **(D)** Proliferative capacity of adherent DKO (blue) and DKO RAS (red) cells continuously exposed to different concentrations of taurine for three days. **(E)** DKO RAS^V12^ MEFs isolated from methylcellulose respond to taurine and muscimol by forming protrusions comparable to DKO cells (S8 and S9 Videos), whereas DKO RAS^V12^ p53kd MEFs isolated from methylcellulose did not respond to taurine (S10 Video).

During the Regulatory Volume Decrease (RVD) following hyposmotic shock, cells can compensate excessive water uptake by forming voluminous protrusions, and our hits LRRC8a, Moesin, Arpc1b, Actr2, as well as taurine and a yet unidentified anion channel have been implicated in this response (Table 1) [11,17,19,20,21]. Because loss of cell volume may hinder cell growth and proliferation [11,12], we investigated whether taurine-evoked chloride currents through GABA_A_ receptor channels cause seclusion of volume in protrusions. We confirmed that DKO MEFs exposed to hyposmotic shock formed protrusions that accumulated volume (Fig 4A; S1 Video), and a similar response was seen in adherent DKO cells exposed to taurine (Fig 4B; S2 Video) and muscimol (S3 Video). Per cell, 1–3 voluminous protrusions appeared to extend from pseudopod membranes (Fig 4C and 4D). The volume secluding effect of taurine was prevented when cells were co-exposed to the general GABA_A_ receptor antagonist Gabazine or the GABRA5-specific antagonist L655,703, or by expression of RAS^V12^, which represses GABRA5 (Fig 5A; S4–S6 Videos). L655,703 treatment or expression of RAS^V12^ also attenuated the formation of protrusions following hyposmotic shock (Fig 5B; S7 Video). Taurine elicited weak tonic currents (40 pA) in DKO MEFs during patch clamp measurements (Fig 5C). These results show that taurine-activated GABRA5-containing GABA_A_ receptor complexes on fibroblasts cause seclusion of volume in membrane protrusions.

Furthermore, we found that GABRA5/B3 expression and volume seclusion by taurine correlated with reduced proliferation. In contrast to DKO RAS^V12^ cells, attached GABRA5/B3-expressing DKO MEFs formed membrane protrusions in response to taurine (Fig 5A) and displayed a dose dependent retardation of proliferation (Fig 5D). Upon loss of anchoring, DKO RAS^V12^ cells arrested and induced GABRA5 and GABRB3 (Fig 3B). When these cells were isolated from methylcellulose, both taurine and muscimol provoked protrusions comparable to DKO cells (Fig 5E; S8 and S9 Videos). In contrast, anchor-deprived fully transformed DKO RAS^V12^ p53kd MEFs, which did not induce GABRA5 and GABRB3 upon loss of anchoring (Fig 3B), did not form protrusions in response to taurine (Fig 5E; S10 Video). Thus, volume seclusion by taurine signaling through GABA_A_ receptors operates as an anti-proliferative mechanism in anchor-deprived conditions.

### GABRA5 expression and taurine production from CoA in human cholangiocarcinomas prolongs survival

**Fig 6:**
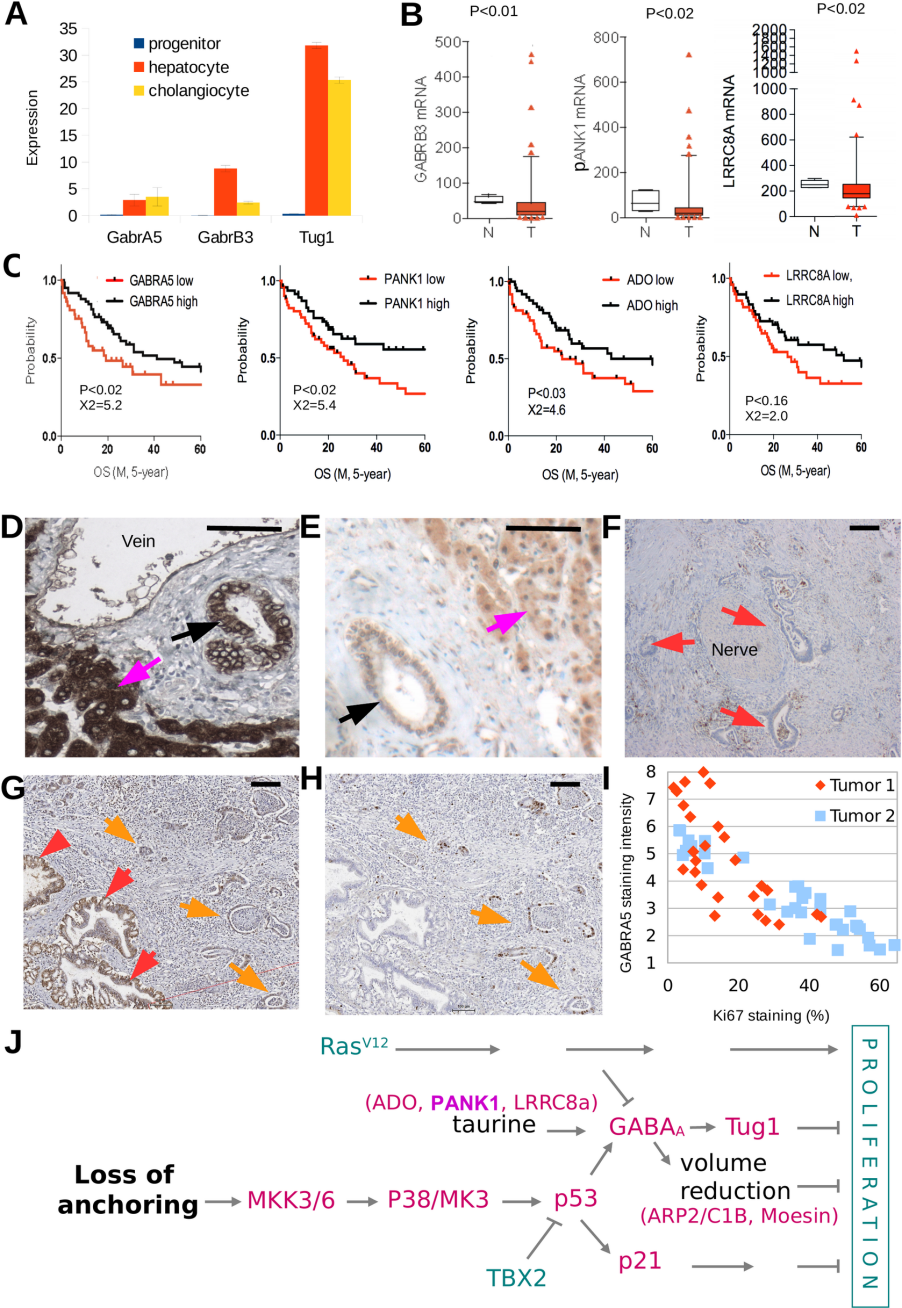
Taurine signaling via GABRA5 affects survival of cholangiocarcinoma patients. **(A)** Expression of GABRA5, GABRB3 and TUG1 in expanding liver progenitor cells (blue), differentiated hepatocytes (red) and cholangiocytes (yellow) (Shin et al., 2011). **(B)** Decreased mRNA expression of GABRB3, PANK1 and LRRC8a in 104 cholangiocarcinomas (red) compared to normal bile ducts (white). **(C)** Survival of 104 patients is related to the expression of GABRA5, PANK1, ADO and LRRC8A in cholangiocarcinomas. Cholangiocarcinomas were classified as “higher than median RNA expression” (black line) or “lower than median RNA expression” (red line) and Kaplan Meyer curves were generated for both groups. **(D)** GABRB3 detected by IHC in hepatocytes (magenta arrow) and cholangiocytes in bile ducts (black arrow). **(E)** GABRA5 detected by IHC in hepatocytes (magenta arrow) and cholangiocytes in bile ducts (black arrow). **(F)** Cholangiocarcinoma lacking GABRB3 protein expression (red arrows) in neoplastic ductules surrounding a nerve fiber. **(G)** Heterogenous expression of GABRA5 in a cholangiocarcinoma that displays high expression in some ductular structures (red arrows), and low expression in other cells (orange arrows). **(H)** Cells that express low levels of GABRA5 often express Ki67 (orange arrows). **(I)** Scatter plot showing an inversed relation between Ki67 and GABRA5 staining in tubular structures from two cholangiocarcinomas. **(J)** We propose that, in addition to p21 induction, tumor suppression involves the formation of cellular protrusions caused by taurine-activated GABA channels to reduce volume of the cell body. This mechanism is the outcome of a balance between the Ras and p38 pathways. The suppression of signaling though GABA channels by Ras^V12^ under adherent conditions is overruled by a p38 stress response that is activated upon loss of anchoring. Proteins indicated in green promote proliferation; genes indicated in magenta inhibit proliferation.

By analyzing microarray data from a murine model for regeneration of the liver [22], where taurine concentration is high, we found more than 15-fold induction of GABRA5 and GABRB3 expression and two-fold induction of TUG1 in non-proliferative cholangiocytes or hepatocytes compared to expanding progenitor cells (Fig 6A). Apparently, in these cell types an inverse correlation exists between the efficacy of GABA_A_ signaling and proliferative capacity. Therefore, we studied the role of GABA_A_ receptors and enzymes involved in taurine supply during transformation of cholangiocytes in human bile ducts (cholangiocarcinoma). PANK1 (Pantothenate kinase) catalyzes the rate-limiting step in the production of CoA, whose stepwise breakdown results in cysteamine that is finally converted into taurine by ADO. LRRC8a is involved in taurine excretion. Microarray-based expression profiling of 104 resected human cholangiocarcinomas [23] revealed reduced expression of GABRB3, PANK1, and LRRC8A, but not GABRA5 or ADO, compared to micro-dissected bile duct controls (Fig 6B, Figure A in S5 Fig). Stratifying the cohort of 104 cholangiocarcinomas for RNA expression above and below the mean revealed that low RNA expression of GABRA5, LRRC8a, ADO and PANK1, but not GABRB3 (Figure B in S5 Fig), was associated with reduction of the median survival time of patients by 1.8, 2.1, 3.3, and 4.3 years, respectively (Fig 6C).

To correlate taurine signaling and proliferation, we determined GABRB3, GABRA5 and Ki67 protein levels in 7 cholangiocarcinoma samples by immunohistochemical staining (IHC). GABRB3 and GABRA5 were detected in hepatocytes and most cholangiocytes (Fig 6D and 6E). In all samples GABRB3 was reduced or completely lost in tumor cells (Fig 6F), whereas GABRA5 protein was reduced in part of the neoplastic ductular structures in 3 out of 7 cholangiocarcinoma samples (Fig 6G). To determine the proliferative activity of the tumors, we stained for the proliferation marker Ki67 (Fig 6H). Only the 3 tumors with reduced GABRA5 protein levels displayed neoplastic ductular structures in which >5% of cells expressed Ki67. In two hyperproliferative tumors, that contained neoplastic ductular structures in which >30% of the cells expressed the proliferation marker Ki67 (Fig 6H) we compared Ki67 and GABRA5 expression levels in individual ductular structures, and found that reduced GABRA5 protein levels strongly correlated (r = -0.84) with increased proliferation (Fig 6I). This strong correlation was confirmed by analyzing gene expression data from cholangiocarcinomas present in the cancer genome atlas (TCGA) (Figure C in S5 Fig), suggesting that taurine signaling through GABRA5 prolongs survival by suppressing cell proliferation. Taken together, these observations are suggestive for a tumor suppressive role of taurine-responsive GABA_A_ receptors in a human cancer type (Fig 6J).

## Discussion

Screening of genome-wide viral shRNA libraries is a powerful method for finding tumor suppressor genes [24]. However, three issues hamper effective screening of shRNA libraries. First, shRNA-mediated interference often causes non-specific transcript destruction. Micro-array experiments have shown that a single RNAi molecule can destroy many different mRNAs [25,26,27]. Off-target silencing complicates the identification of functional transcripts and calls for testing multiple vectors to exclude false positives. Secondly, the transcriptome is more dynamic and complex than previously anticipated, including transcript variants and many types of non-coding RNAs that are missing in current libraries [28,29]. Lastly, genome-wide loss-of-function screening is challenging due to the high complexity of the libraries.

To avoid these hurdles, we have validated a method to produce tailor made low complexity LEGO shRNA libraries that focus on transcripts that are repressed during a phenotypic transition between two cellular counterparts (*i*.*c*., transformation). Low complexity LEGO shRNA libraries are enzymatically produced after subtracting the transcriptomes of the two phenotypic counterparts, which offers several advantages. Their complexity is reduced by the exclusion of vectors targeting the majority of the genome that is not expressed. In addition, subtractive hybridization causes normalization of the transcriptome and enrichment for the repressed transcripts suspected to control the phenotypic transition. Importantly, by focusing on relevant genes, false positive background is reduced. Furthermore, numerous shRNA vectors are enzymatically produced per selected transcript. Therefore, the identification of suppressor transcripts relies of multiple shRNA vectors, further reducing false positives due to off-target silencing. Moreover, the efficacy of these shRNA vectors varies (Figure J in S1 Fig), allowing the selection of knockdown levels optimal for the phenotypic condition under study. Finally, LEGO libraries allow studying all polyadenylated transcripts independent of genome-annotation.

To identify suppressors of anchorage-independent growth, we produced and screened two LEGO libraries enriched up to 20-fold for vectors targeting transcripts repressed upon transformation by TBX2 expression (LEGO-TBX2) or p53 knockdown (LEGO-p53kd). Our method was validated by the identification, without false positives, of several known coding and non-coding p53 pathway players, including TUG1 and mir-34a, which were reported to be direct transcriptional non-coding targets of p53 [13,14]. The benefit of preselecting targets by transcriptome subtraction is illustrated by the identification of 35 suppressor genes that were repressed in the transformed genotypes. We found that p21 induction upon anchor deprivation of non-transformed MEFs relies on induction of the downstream p38 effector kinase MK3. Our data support the hypothesis that transformation can be achieved by attenuating stress signaling through the MAP kinase p38, which in turn diminishes p53 pathway activation [30,31,32,33,34].

In addition, we revealed a novel anti-proliferative mechanism, which involves activation of GABA_A_ receptors by taurine produced from CoA breakdown. Taurine is abundant in tissues, the bulk residing intra-cellularly due to active uptake by a transporter protein (Taut). However, it is released when cells are stressed by hypoxia, ischemia, hypoglycemia, free radicals, or hyposmotic shock [11,35,36,37,38,39]. Taurine is considered the primary organic osmolyte involved in the Regulatory Volume Decrease (RVD) following hyposmotic shock, which involves the efflux of anions and organic osmolytes to maintain cell volume [11,40,41,42]. While volume reduction by taurine is believed to primarily result from its osmolytic properties, we here show for the first time that taurine can activate GABA_A_ receptors on MEFs to seclude volume in protrusions. Similar protrusions were observed in 3T3 fibroblasts, in renal CD8 cells, and in Rat-1 fibroblasts during the RVD upon hyposmotic shock [17,19,40]. Taurine was shown to be a potent agonist of GABRA4-containing GABA_A_ receptors on neurons [14]. GABA_A_ receptors are chloride channels well known for their involvement in neurotransmission in the nervous system, but their function on fibroblasts remained unnoticed. In MEFs, protrusion formation by taurine depended on GABRA5 and correlated with reduced proliferation both in attached and anchorless cells.

The inclusion of GABRA5 (and GABRB3) in the receptor complex is affected by p53, RAS^V12^, transformation, differentiation, and loss of anchoring. We propose that seclusion of volume in pseudopodial protrusions by taurine-activated GABRA5 subunits reduces the effective volume of the cell body and counteracts cell growth required for sustained proliferation (Fig 6J). Alterations of cell volume are key events during cell proliferation, which eventually requires an increase of cell volume, and apoptosis, which typically involves cell shrinkage [10,11,12]. Protrusion formation results from activation of RAC and CDC42, which control the creation of the actin meshwork by the ARP2/3 complex and Moesin [17,19,43]. Our screen identified two members of the ARP2/3 complex and Moesin as suppressors of anchorless proliferation, suggesting that protrusion formation *per se* suppresses anchorless proliferation *in vitro*.

We obtained *in vivo* evidence for deregulation of this tumor suppressive mechanism in human cholangiocarcinomas [21], where several genes involved in taurine-mediated volume regulation (*GABRB3*, *PANK1* and *LRRC8a*) were repressed. Reduced mRNA levels of *GABRA5*, *ADO*, *PANK1* and *LRRC8a* in tumors correlated with shortened survival of patients, and reduced GABRA5 protein expression correlated with increased proliferation. GABA_A_ receptor activity has been implicated in proliferation and tumorgenesis before: it was reported to reduce proliferation of neural crest stem cells [44], pancreatic ductal adenocarcinoma [45], neuroblastoma [46], and medulloblastoma [47]. Research in additional human tumors and mouse models is needed to provide further evidence for the tumor suppressive activity of taurine-mediated GABA_A_ receptor signaling *in vivo*.

## Materials and methods

### cDNA synthesis

RNA was isolated using Rneasy columns (Qiagen) and DNAse treated. For subtractive hybridization, cDNA was produced using the SMART cDNA synthesis method (Clonetech) and amplified using the Expand polymerase (Roche) during 20 PCR cycles in 45 separate 50 μl reactions. After PCR, the cDNA was precipitated before purification on YM100 columns (Microcon). For quantitative PCR, cDNA was produced using the Superscript 2 reverse transcriptase.

### Subtractive hybridization

cDNA was produced from non-transformed (tester) and transformed (driver) cells residing 4 days in methyl cellulose. One μg of tester cDNA preparation was *Alu*I digested and ligated in 20 μl buffer 4 (NEB), supplemented with ATP (1 mM), and either Adapter A or B (20 μM). After incubation with *Alu*I (5U; 1 h at 37 ^o^C), ligase (5U) was added to the reaction, which was incubated overnight in a thermocycler cycling between 30 ^o^C (10 s) and 10 ^o^C (10 s). After heat inactivation of the enzymes (20 min at 80 oC), the samples were filtered twice to remove excess Adapters and concentrated (20 ng/μl tester A and B) using a YM 100 column (Microcon). Two μg driver cDNA was digested overnight in 40 μl buffer 4 (NEB) using *Alu*I (10U), heat inactivated (20 min 80 ^o^C), and concentrated to 200 ng/μl using a Microcon YM 10 column.

For optimal subtractive hybridization, 10 ng Adapter A and B ligated tester fragments were separately mixed with 600 ng of driver fragments. In a volume of 5 μl covered by mineral oil, containing PEG (6%; MW 6/8000), NaCl (0.5 mM), Hepes (100 mM), fragments were melted (3 min 95 ^o^C) and subsequently rehybridized for 45 h at 68 ^o^C. Then, the Adapter A and B ligated samples were mixed, 1.25 μl PEG 55% was added and hybridization was continued for 24 h. After addition of 250 μl of dilution buffer (50 mM NaCl, Hepes 100 mM) and incubation at 68 ^o^C for (10 min), 100 ng of streptavidin-coated magnetic beads (Dynalbeads) in 250 μl dilution buffer were added (10 min, room temperature (RT)). Biotinylated Adapter B ligated fragments were pulled down using a magnet, washed twice with dilution buffer (300 μl, 5 min, 68 ^o^C), resuspended in 100 μl *pfu* polymerase mix (Invitrogen) containing only dNTPs (10 mM each). The reaction was hotstarted by adding *pfu* polymerase (1U) at 75 ^o^C to fill in the Adapter sequences (5 min), and stopped by the addition of EDTA (10mM). The beads were recovered using a magnet, cooled, and washed twice with NaOH (100 nM), and once in Tris buffer (10 mM). Half of the beads were used as a template for nested PCR using *pfu* polymerase. The first PCR reaction (1000 μl *pfu* PCR mix divided over 40 separate 25 μl reactions) used primer PCR1 (30 cycles; 15 s 94 ^o^C; 30 s 64 ^o^C; 90 s 94 ^o^C). The 40 reactions were pooled, and 2 μl was used as a template in the secondary PCR (1000 μl *pfu* PCR mix divided over 40 25 μl reactions) using the nested primers PCR2a and PCR2b (16 cycles; 15 s 94 ^o^C; 30 s 64 ^o^C; 90 s 94 ^o^C). Primer PCR2a was biotinylated for further applications. After PCR, the subtracted library was precipitated and purified on a YM100 column (Microcon).

### Validation of subtracted libraries

The composition of the subtracted libraries was determined by dotblot analysis. A panel of 15 genes were spotted onto a membrane and hybridized with radiolabelled subtracted libraries or cDNA. The strength of the radioactive signal on the dotblot was quantified by phospho-imaging.

### Construction of RNAi libraries

Restriction sites on the Adapter A were used to process the selected sequences into 19–20 bp inverted repeats, which were ligated into a pRETRO SUPER vector. Ten μg of the subtracted library was processed in 100 μl buffer 4, containing SAM (1 mM), and the looped Adapter C. One hour after addition of *Mme*I (5U), ligase (1U) was added overnight. After heatinactivation (80 ^o^C for 15 min), NbBC IA (5U) was added during 1 h. On ice, streptavidin-coated magnetic beads (100 ng) were used to pull down the biotinylated primer PCR2a. After the supernatant was replaced by 100 μl buffer 2 (NEB) containing primer 3 (100 nM), and dNTPs (10 mM each) the sample was heated (70 ^o^C for 20 min) to inactivate the enzyme and release the nicked hairpins from the beads. Exo- Klenow polymerase (10 U) was added to elongate the primer 3 (1 h at 37 ^o^C) and to form an inverted repeat. *Bpm*I (10 U) digestion (1 h at 37 ^o^C) removed the duplicated Adapter A ends, generating 91 bp fragments with CT overhangs on both sides. The fragments were separated (4.5% agarose gel) and 91 bp fragments were isolated from the gel by freeze squeezing and phenol extraction. The fragments were ligated into a modified pSUPER RETRO vector, and the ligation mix was introduced into *Eschericha coli*. Plasmids from approximately 150,000 individual small colonies were isolated by maxiprep (Genomed). One μg was digested with *Bsg*I to remove an obsolete part of Adapter C, and the 6834 bp vector fragment was isolated from gel (Qiagen). After religation of the vector, the plasmids were used to transform bacteria. The plasmids from 150,000 individual small colonies were pooled and a maxiprep (Genomed) was performed to isolate plasmids.

For mobilization, the selected shRNA inserts were amplified from cells together with the H1 promotor and flanking recombination sites using the primers listed in Figure D in S1 Fig and reinserted in pRETRO Super backbones using the BP clonase (Invitrogen).

### Retroviral infections

Phoenix cells were transfected with 16 μg of the desired construct and 4 μg pCL-eco. 48 h and 60 h post transfection, retroviral supernatant was filtered through 0.45-μm filters (mixed cellulose ester membrane; Millipore). MEFs were infected with fresh viral supernatants supplemented with Polybrene (4 μg/ml). To ensure full coverage of LEGO libraries in host cells, several infections were performed in parallel.

### Quantitative PCR

qPCR to measure p21, HPRT, miR-34a, MK3, and CAMA2 transcripts was performed using the light cycler (Roche) as described by the manufacturer, using the primers listed in Figure C in S1 Fig.

### Cell culture in methylcellulose

We previously described the culture of *Rb*^*-/-*^*107*^*-/-*^ or DKO MEFs and DKO RAS^V12^ MEFs [2,3]. For methylcellulose assays, the number of MEFs indicated were suspended in 5 ml 1.3% methylcellulose (Sigma) solution in GMEM supplemented with fetal calf serum at a concentration of 10%, non-essential amino acids (Invitrogen), pyruvate (Invitrogen), and penicillin/streptomycin (Invitrogen) and plated on six-well ultra-low-attachment surface plates (Corning Incorporated). Colony formation was analyzed after two weeks using Gelcount (Oxford-Optronix). Cells were isolated from methylcellulose by diluting 5 ml methylcellulose containing the cells in 45 ml ice-cold phosphate-buffered saline (PBS) (Gibco), followed by centrifugation (15 min) and aspiration of methylcellulose-PBS. To retrieve colonies, cells were spun at 500 rpm; to retrieve colonies plus single cells, cells were spun at 2000 rpm. To test the p38 inhibitor SB20358 (10mM) and taurine (500mM), cells were suspended in 2.5 ml methylcellulose in snap-cap tubes (DB falcon) and fresh SB20358 or taurine was mixed in every other day. ADO knockdown cells were pretreated with taurine for 3 days before they were suspended in methylcellulose.

### Gene expression profiling

Gene expression profiling of cells was performed on two platforms as described by the manufacturers (NKI and illumina).

### Pathway analysis

Pathway and biological functional category analysis was performed using the Ingenuity Pathways Analysis program (Ingenuity IPA 6.3−1402).

### Amplification of shRNA inserts for deep sequencing

For quantification by deep sequencing, shRNA inserts were amplified from cells using the primers listed in Figure D in S1 Fig and sequenced using the Hiseq system (Illumina) as described by the manufacturer.

### Identification of shRNA targets and selection of the suppressor transcripts

The transforming shRNA vectors selected from LEGO libraries during a proliferation assay in methyl cellulose are identified by deep sequencing. shRNA insert sequences were extracted from deep sequencing data by identification of complementary stem-loop-stem sequences (1 mismatch due to a PCR artifact/sequence error was allowed). Copy numbers of shRNA inserts before and after either round of selection were normalized between experiments. shRNA vectors enriched >3-fold were considered to be transforming shRNA vectors. Potential shRNA target mRNAs were identified from the mouse NCBI nucleotide database (version May 2009, sequences with >500 AluI sites removed) by blast search. Redundancy among retrieved nucleotide sequences was reduced by clustering of the sequences using BLASTClust. Redundant sequences with 95% sequence identity and 90% sequence coverage (single-sided) were combined and considered to represent a single transcript. A minimal set of clusters that explains all observed shRNA tags was established according to maximum parsimony using a breadth-first approach. Sequence clusters with a >15% coverage of *Alu*I sites were considered for further analysis. A sequence cluster was omitted from the result list when it included different transcripts derived from multiple gene copies, because this hinders unambiguous identification of the true target transcript.

In a final bioinformatics step, the transcripts targeted by multiple selected transforming vectors are are identified in the NCBI nucleotide database to obtain a suppressor transcript profile for anchorless proliferation. The absolute number of transforming vectors formed by each transcript was counted. A transcript was classified as a suppressor transcript when it was targeted by a total of at least three different transforming shRNA vectors selected from LEGO-TBX2 and at least 3 selected from LEGO-p53kd. Furthermore, the number of shRNA vectors that can potentially be produced from a suppressor transcripts when *Alu*I sites are maximally used during the enzymatic procedure was deducted, and the percentage that resulted in a transforming vector was calculated. Transcripts were ranked by multipliing the percentage of transforming vectors and the absolute number of transforming vectors selected during screening.

### Western blotting

Cells were lysed for 30 min on ice in lysis buffer containing 150 mM NaCl, 50 mM HEPES (pH 7.5), 5 mM EDTA, 0.1% NP-40, 5 mM NaF, 0.5 mM vanadate, 20 mM β-glycerolphosphate, and 1 tablet complete protease inhibitor cocktail (Roche) per 50 ml. After centrifugation, protein concentration was determined using Bio-Rad protein assay. For immunoblot analysis, 20–30 μg protein was separated on 12% SDS-polyacrylamide gels. Blotting was performed by using standard protocols.

### Immunohistochemistry

Cholangiocarcinomas (n = 7) were stained using GABRA5, GABRB3, or Ki67 antibodies (LifeSpan Biosciences). Five-micrometer sections of paraffin-embedded liver tissue were deparaffinized in xylene, and rehydrated in an ethanol to water series. Heat-induced antigen retrieval was performed with 10 mM citrate buffer (pH 6.0) or 10 mM Tris with 1 mM EDTA (pH 8.0) at 98 ^o^C in a water bath, followed by cooling at RT for 30 min. Antigen retrieval by enzymatic digestion was performed with proteinase K (Dakocytomation, Glostrup, Denmark) for 10 min at RT. Dual endogenous enzyme block (Dakocytomation) was used (10 min RT) to quench endogenous peroxidase activity, and background staining was blocked 30 min with 10% normal goat serum (Sigma-Aldrich, St.Louis, US). Sections were incubated with the labeled secondary antibody Envision (Dakocytomation) for 1 h at RT. The signal was developed in 0.06% 3,39-diaminobenzidine (DAB) solution (Dakocytomation). Replacement of primary antibody with washing buffer served as negative control. All tissues were stained in batch per antibody to avoid technical differences. Stainings were digitalised for analysis using scanscope (Aperio).

### Imaging of Regulatory Volume Decrease

MEFs plated on poly-D-lysine-coated glass cover slips were loaded with 5mM Calcein-AM (Molecular Probes) for 30 min at 37 ^o^C and transferred to recording chamber, equipped with a microscope for time lapse photography as described previously (Ridder et al., 2011). The recording chamber was continuously perfused (5 ml/min) with (isosmotic) ACSF (pH 7.4), consisting of 125 mM NaCl, 3 mM KCl, 1.2 mM NaH_2_PO_4_, 2.4 mM CaCl_2_, 1.3 mM MgSO_4_, 25 mM NaHCO_3_, 10 mM glucose, and carboxygenated in 5% CO_2_−95% O_2_. Images were taken every 25 s. To record the effect of hyposmotic shock, cells pretreated with isosmotic ACSF were perfused with hyposmotic solution that had a 40% reduced osmolarity as compared to ACSF. In addition, the effects of (combinations of) taurine (500 mM), muscimol (100 mM), gabazine (10 mM), and L655,708 (100nM) were determined.

### Electrophysiology

For electrophysiological recordings we made use of Clampex 9.2 along with a 700B amplifier and a 1322A digitizer (all from Molecular Devices, Sunnyvale, California, USA). Cell were identified and patched using an Olympus BX50WI DIC microscope with a 40x, 0.8nA W objective (all Olympus corporation, Tokyo, Japan). Data was acquired using an internal 4 pole Bessel filter (3 kHz) and a sample frequency of 20 kHz. Cells were patched using electrodes of 2.5–4 MΩ resistance. The intracellular solution contained: 70 mM Cs—Gluconate, 70 mM CsCl_2_, 10 mM HEPES, 0.5 mM NaGTP, 5 mM Mg_2_-ATP, 10 mM EGTA and 10 mM K-Phosphocreatine (pH 7.3, 290 Osm).

## Supporting information

S1 Table Enrichment for shRNA vectors targeting suppressors of anchorless proliferation in LEGO libraries. The percentage of genome wide transcripts that are reduced to various extends in anchorless proliferating transformed DKO RAS^V12^ TBX2 or p53kd cells compared to their arrested non-transformed DKO RAS^V12^ counterparts is shown in the first two rows. The next two rows show the percentage of these reduced transcripts targeted by the LEGO-TBX2 and LEGO-p53kd libraries. The final two rows show the fold enrichment for these transcripts in the LEGO libraries.
(PDF)

S2 Table Suppressors of anchorless proliferation. Seventy-nine genes involved in suppression of anchorless proliferation are listed. Retesting of 15 selected genes using newly-designed shRNA vectors (marked *) supported their suppressive role. The suppressor transcripts they encode were ranked according to the score obtained by multiplying the absolute number of selected transforming vectors that targeted this transcript and the percentage this number represents of the maximal number of shRNA vectors potentially synthesized using each *Alu*I site present in this transcript (Also see S1I Fig). In addition, its induction in DKO RAS^V12^ MEFs after loss of anchoring, its repression by RAS^V12^ expression, and its repression in anchorless DKO RAS^V12^ TBX2 or DKO RAS^V12^ p53kd cells compared to anchorless DKO RAS^V12^ cells are shown. Genes reduced >1.66 fold in anchorless DKO RAS^V12^ TBX2 or DKO RAS^V12^ p53kd cells are shown in red, genes induced >1.66 fold in DKO RAS^V12^ after loss of anchoring are shown in green; genes reduced >1.66 fold in attached DKO RAS^V12^ compared to DKO cells are shown in magenta. They included 35 genes that were repressed (>1.66 fold) either in anchorless DKO RAS^V12^ TBX2 cells (n = 28), in DKO RAS^V12^ p53kd cells (n = 33), or in both fully transformed genotypes (n = 26). Note that 13 genes are regulated by anchoring, RAS^V12^, p53 and TBX2. For each suppressor transcript, the accession number is shown and whether it encodes for a protein. Genes linked to taurine induced volume reduction are shown in red. For transcripts marked # Q-PCR was performed to validate expression data. Intriguingly, 4 transcripts encoding Cathepsins (A, B, D, L) were identified, suggesting these proteases play an important role in the suppression of anchorless growth. Furthermore two pairs of genes lying side by side in the genome were identified (*Ctsa*/*Ptlp* and *Ptrf*/*Stat3*).
(PDF)

S1 Fig Optimization of subtractive hybridization. **A. Schematic representation of adaptors and primers used for subtractive hybridization**.
For the production of LEGO libraries, we designed a set of oligonucleotide adapters A, B, and C that allow (1) PCR-based suppression subtractive hybridization (SSH) (adapted from ref. 7), and (2) enzymatic processing of selected fragments into shRNA vectors. For SSH, adapters A and B contain two priming sites for a first round of PCR (dark blue) and nested PCR (light blue and yellow). Adaptor A and B lack a 5' phosphate group to prevent ligation of the antisense adaptor strand to the cDNA. In addition, part of adapter A participates in library processing (red) using recognition sites for *Mme*I (violet), *Bpm*I (orange) and N*bBC*IA (green). The phosphorylated adapter C has a degenerate 2 nt overhang, contains the 9 nt sequence destined to form the loop of the shRNA molecules (purple), and forms two *Bsg*I sites (pink) upon synthesis of the complementary strand. The adapter for pRETRO Super modification is inserted into pRETRO Super behind the H1 promotor using *Bgl*II and *Hind*III sites. Subsequent *Bsg*I (pink) digestion of the adapted pRETRO Super creates two CT overhangs (red): one that follows the transcription initiation signal of the H1 promotor (brown; CCCC) and the other in front of the transcription termination signal (dark green; TTTTT). This allows insertion of the shRNA inserts produced by our protocol, which have AG overhangs after *Bpm*I digestion. These overhangs are determined by the *Alu*I digestion that precedes adaptor A ligation: the cDNA ligated to the adapter A has blunt CT ends as a result of the *Alu*I site (= AGCT), which are transformed in the AG overhangs as *Bpm*I cuts exactly here during digestion of SSH PCR product (also see S1I Fig; step 7). The G in front of the CT overhang (pale yellow) reconstitutes part of this *Alu*I restriction site, and causes the transcribed shRNA molecules to start with GCT.
**B. Sequences of the adapters and primers used for subtractive hybridization**.
Note that the first PCR uses only one primer recognizing a priming site present on both Adapter A and B (dark blue). Klenow Primer 3 serves as a primer for Klenow polymerase. Primers used for deep sequencing of shRNA inserts can be given an 8 nt barcode (green) and sequenced all together. Attb1 and attb2 recombination sites required for gateway cloning are shown in red.
**C. Schematic representation of PCR-based SSH used to select sequences for LEGO library production**.
The subtraction and shRNA production procedure was optimized using a pair of isogenic embryonic stem cell lines, H- (called driver cDNA) and H+ (called tester cDNA), the latter expressing the Hygromycin B resistance gene hyg. (1) Polyadenylated RNA isolated from tester (H+; red) and driver (H-; light blue) cells is converted to cDNA and digested with *Alu*I. 5’ ends (not 3’ ends) of *Alu*I-digested tester cDNA are ligated to either Adapter A (1a) or B (1b) in separate reactions. (2) The Adapter A- and B-ligated tester fragments are hybridized separately to an excess of *Alu*I-digested driver cDNA. As an adaptation to the original protocol (Diatchenko et al., 1996), we prolonged this this first hybridization step until equilibrium was reached. Due to the second order kinetics of hybridization, which causes the more abundant fragments to re-anneal faster, an equal number of each of the AluI fragments is expected to remain single stranded at equilibrium (also see Figure D in S1 Fig). Moreover, the ratio of adaptor-ligated to adaptor-less single-strands at equilibrium depends on the relative expression levels in the tester and the driver cells: more single strands will carry an adapter if the corresponding RNA was more abundant in the tester cells than in the driver cells, and vice versa.
(3) Subsequently, the Adapter A- and B-containing samples were mixed, and further hybridization of remaining single strands was enforced by adding the volume exclusion agent polyethylene glycol (PEG). In this step, hybrids carrying both the Adapter A and B at their ends can form. Again, their abundance depends on the relative expression levels in the tester and the driver cells.
(4) After filling in the complementary adapter strand, these hybrids can be exponentially amplified by nested PCR. Amplification of hybrids carrying two identical adapters is suppressed by intrastrand folding (to prevent intrastrand folding, the sites required for subsequent enzymatic processing of LEGO libraries are only present on adaptor A and not on adaptor B). The PCR product will be enriched for fragments that were overrepresented in the tester cell line H+ and contain normalized and reduced numbers of equally and less expressed fragments, respectively.
**D. Schematic overview of the kinetics of re-annealing during subtractive hybridization**.
After mixing the driver and tester cDNA fragments, the strands are melted and allowed to re-anneal. Adapter A and B ligated fragments are hybridized to the driver cDNA in separate reactions. At the start of re-annealing, the different single-stranded *Alu*I fragments are present at different concentrations because of different expression levels in the cells. Re-annealing follows second order kinetics. This means that more abundant cDNA fragments, which meet more often, re-anneal faster than scarce cDNA fragments, until an equilibrium concentration is reached (colored lines). At equilibrium, a normalized number of each different *Alu*I fragment present in the mixture is expected to remain single stranded (black line). After the first hybridization has reached equilibrium, adapter A and B ligated samples are mixed. To stimulate further re-annealing, the volume exclusion agent PEG is added to cause molecular crowding increasing the effective concentration.
**E. Quantification using spotblots**.
To determine the success of subtractive hybridization, the abundance of a panel of 9 genes was determined in the *Alu*I digested tester cDNA and the subtracted library. Library and cDNA were radiolabelled and hybridized to a nitrocellulose filter on which the differentially expressed *hyg* gene, the abundant *actin*, *tubulin*, and *E cadherin* genes and the lower expressed *Msh6*, *p53*, *CDK1* and *Cyclin A* genes were spotted. In tester cDNA, *actin*, *tubulin* and *E cadherin* transcripts were abundant, while *Msh6*, *p53*, *CDK*1, *Rb*, *Cyclin* A and *hyg* transcripts were scarce. In de subtracted cDNA library, all fragments were normalized to low equal levels, while the *hyg* fragments were strongly enriched.
**F. Optimization of enrichment by subtractive hybridization: hybridization time**.
Normalization and enrichment improved by prolonged duration of the first hybridization. We first determined the time required to reach equilibrium for optimal normalization during the first hybridization and examined the effect of PEG addition. Adapter A and B-ligated tester H+ cDNA preparations were separately mixed with a fixed amount of driver H- cDNA at (ratio of 1:35) and the first hybridization was allowed to proceed from 0 to 45 hours in the presence of 5% PEG. Subsequently, the Adapter A- and B-containing samples were mixed and the PEG concentration was raised to 15%. After the second hybridization period (24 h), the subtracted libraries were amplified by PCR and the abundance of a panel of 9 gene sequences was determined. To measure the abundance of different genes in the subtracted libraries, PCR products were radiolabelled and hybridized to a nitrocellulose filter on which a panel of 9 genes was spotted. After 45 hours, the abundant *actin*, *tubulin*, *E cadherin* and *Msh6* genes and the lower expressed *p53*, *CDK1*, *Rb* and *Cyclin A* genes, which are equally expressed in the two cell lines H+ and H-, were reduced to normalized low levels in the subtracted library, but not completely removed. In contrast, *hyg* sequences, which are specific to the tester cDNA, were strongly enriched.
**G. Optimization of enrichment by subtractive hybridization: ratio tester/driver**.
Subsequently, we determined the effect of the amount of driver cDNA added to the first hybridization on the efficacy of subtraction. The procedure was followed using increasing amounts of driver H- cDNA during the first hybridization. As expected, normalization and enrichment improved when increasing amounts of driver cDNA were added to the reaction.
**H. Optimization of enrichment by subtractive hybridization: polyethyleneglycol**.
The addition of PEG during the second hybridization was essential for optimal subtractive hybridization. Adapter A- and B-ligated tester H+ cDNA preparations were separately mixed with a fixed amount of driver H- cDNA at (ratio of 1:35) and the first hybridization was allowed to proceed from 0 to 45 hours in the presence of 5% PEG. Subsequently, the Adapter A- and B-containing samples were mixed with or without raising the PEG concentration to 15%. After the second hybridization period (24 h), the subtracted libraries were amplified by PCR and the relative abundance of *hyg* was determined. Without addition of PEG, subtractive hybridization was less effective and no subtracted libraries could be amplified when the first hybridization period proceeded more than 18 hours.
In conclusion: We found the efficacy of subtractive hybridization to be highest by adding 60-fold excess of driver cDNA to the first hybridization reaction and by allowing this step to proceed for 45 h (S2F and S2G Fig). Importantly, the addition of PEG during the second hybridization step was crucial for optimal subtractive hybridization: it increased enrichment of *hyg* sequences more than 12 fold (S2D Fig).
**I. Enzymatic production of shRNA vectors from subtracted libraries**.
Using restriction sites located on the adaptor A flanking the SSH PCR product (the subtracted cDNA library), and on adaptor C, the selected cDNA sequences can be processed into inverted repeats and inserted into pRETRO Super vectors to produce the subtracted retroviral LEGO shRNA library. Because adaptor A is ligated to both ends of each *Alu*I fragment, two shRNA vectors can result from each *Alu*I site. Note that the cDNA parts have AG ends resulting from *Alu*I digestion before adaptor ligation. (1) Digestion with *Mme*I (violet), which cuts outside its recognition site, leaves 18 base pairs of cDNA (blue) with a 2 nt overhang attached to Adapter A. (2) Using its 2 nt degenerate overhang (N_1_N_2_), the looped Adapter C carrying the 9 nt shRNA loop is ligated to the cDNA, forming a short hairpin. (3) After inactivating the ligase, *N*.*Bbvc* IA (green) nicked Adapter A. (4) The botinylated primer Nested PCR2a used to amplify the subtracted library allows isolation of the nicked hairpins using streptavidin-coated beads. (5) Subsequent heating inactivated *N*.*Bbvc* IA and, due to the nick, the hairpins were released from the bead, allowing primer annealing to the now exposed single-stranded region. (6) Primer extension by Klenow DNA polymerase generated double-stranded inverted repeat sequences. (7) *Bpm*I (orange), which cuts 16 bp away from its recognition site, was used to remove the duplicated adapter A ends, releasing inverted repeats with AG overhangs. (8) These are isolated after separation on gel, and (9) ligated behind the H1 promoter (brown) of a modified pRETRO Super backbone with CT overhangs (S1 Fig). (10) Finally, part of Adapter C is removed using *Bsg*I (pink), which cuts 16 bp from its restriction site, creating functional pRetro SUPER LEGO shRNA vectors.
**J. Functionality of shRNA vectors**.
Using our procedure, we generated eleven different vectors from the 998 bp *hyg* open reading frame. To confirm their functionality, the knockdown level they achieved was measured by quantitative PCR. After infection of *hyg*+ cells, 9 vectors reduced *hyg* transcripts by 40 to 80 percent.
(PDF)

S2 Fig Validation of 17 selected hits as suppressors of anchorless proliferation. **A**. Newly designed shRNA sequences inserted into pRETRO Super vectors used for knockdown of identified suppressor transcripts.
**B.** Level of knockdown achieved for a subset of these shRNA vectors in typical experiments as measured by quantitative PCR.
**C.** Retesting of selected hits. Newly designed shRNA vectors targeting 17 selected suppressor transcripts were tested for their effect on proliferation in colony forming assays in methylcellulose.
**D.** miR-34a is a suppressor of anchorless proliferation. Colony forming assays showing increased proliferation of *Rb*^*-/-*^*107*^*-/-*^ RAS^V12^ MEFs in methylcellulose upon knockdown (kd) of the non-coding suppressor transcript miR-34a. Viral co-expression (expr) of miR-34a in *Rb*^*-/-*^*107*^*-/-*^ RAS^V12^ TBX2 MEFs largely reverted transformation.
**E.** The p38 stress pathway suppresses anchorless proliferation. Colony forming assays showing increased proliferation of *Rb*^*-/-*^*107*^*-/-*^ RAS^V12^ MEFs in methylcellulose upon knockdown (kd) of MK3, Map2k6 and Map2k3. Viral co-expression (expr) of MK3 in *Rb*^*-/-*^*107*^*-/-*^ RAS^V12^ TBX2 MEFs largely reverted transformation.
In C, D and F, pictures of wells were taken 3 weeks after 5x10^4^ cells were seeded per well. The Luc vector (Mock) was used as a negative control for miR-34a.
F. Network analysis of hits. Network revealed by Ingenuity analysis using 23 hits that were induced (1.66–7.05 fold) in non-transformed DKO RAS^V12^ cells after loss of anchoring, but repressed (1.66–11.9 fold) in both transformed genotypes during anchorless proliferation.
(PDF)

S3 Fig Induction of suppressor proteins upon loss of anchoring. **A.** Western blots showing strong and weak p21 protein induction in anchor deprived DKO RAS^V12^ and DKO RAS^V12^ TBX2 cells, respectively. Lanes derived from different sections of a single gel are shown.
**B.** GabrA5 and GabrB3 protein levels in attached and non-attached DKO RAS^V12^ and DKO RAS^V12^ p53kd cells. Lanes shown represent different sections of the same gel.
(PDF)

S4 Fig Expression of GABA_A_ receptor subunits. RNA sequencing revealed low expression of particular GABA_A_ receptor subunits in attached DKO RAS^V12^ (blue), in anchorless DKO RAS^V12^ (red) and in anchorless DKO RAS^V12^ p53kd cells (yellow). Subunits GabrB3 and GabrA5 were regulated both by loss of anchoring and by p53. For comparison, their expression was over 325-fold lower than that of Hprt.
(PDF)

S5 Fig GABRA5 and ADO expression in cholangiocarcinomas. **A.** mRNA expression of GABRA5 and Ado did not change in 104 cholangiocarcinomas (red; T) compared to normal bile ducts (white; N) and surrounding liver tissue (green; SL)
**B.** Survival of 104 patients is not affected by the expression of GABRB3 in cholangiocarcinomas. Cholangiocarcinomas were classified as “higher than mean RNA expression” (black line) or “lower than mean RNA expression” (red line) and Kaplan Meyer curves were generated for both groups.
**C.** Scatterplot showing strong negative correlation between GABRA5 gene expression and Ki67 gene expression in 37 cholangiocarcinomas present in the TCGA database (slope = -1,694667415; r.sq = 0,2608221533; p value = 0,0015).
(PDF)

S1 Video Time laps video showing the response of attached DKO MEFs to hyposmotic shock treatment. (AVI)

S2 Video Time laps video showing the response of attached DKO MEFs to taurine treatment. (AVI)

S3 Video Time laps video showing the response of attached DKO MEFs to muscimol treatment. (AVI)

S4 Video Time laps video showing lack of response of attached DKO MEFs coexposed to taurine and Gabazine. (AVI)

S5 Video Time laps video showing lack of response of attached DKO MEFs coexposed to taurine and L655703. (AVI)

S6 Video Time laps video showing lack of response of attached DKO RAS^V12^ MEFs to taurine treatment. (AVI)

S7 Video Time laps video showing lack of response of attached DKO MEFs exposed to hyposmotic shock in the presence of L655703. (AVI)

S8 Video Time laps video showing the response of non-adherent DKO RAS^V12^ MEFs to taurine treatment. (AVI)

S9 Video Time laps video showing the response of non-adherent DKO RAS^V12^ MEFs to muscimol treatment. (AVI)

S10 Video Time laps video showing lack of response of non-adherent DKO RAS^V12^ p53kd MEFs to taurine treatment. (AVI)

## Abbreviations

LEGO: Low-complexity by Enrichment for Genes shut Off
MEFs: mouse embryonic fibroblasts
RVD: Regulatory Volume Decrease
SSH: Suppression Subtractive Hybridization
