# Supplementary material for: RNAi screening of subtracted transcriptomes reveals tumor suppression by taurine-activated GABAA receptors involved in volume regulation
Source: PLoS One. 2018 May 22;13(5):e0196979. doi: 10.1371/journal.pone.0196979 (PMC5963783; doi:10.1371/journal.pone.0196979)
Supplement: S1 Table — The percentage of genome wide transcripts that are reduced to various extends in anchorless proliferating transformed DKO RASV12 TBX2 or p53kd cells compared to their arrested non-transformed DKO RASV12 counterparts is shown in the first two rows. The next two rows show the percentage of these reduced transcripts targeted by the LEGO-TBX2 and LEGO-p53kd libraries. The final two rows show the fold enrichment for these transcripts in the LEGO libraries. (PDF) [file pone.0196979.s001.pdf]

	Expression transcripts genome wide (%)		Expression target transcripts LEGO library (%)		Fold enrichment in LEGO library	
	p53kd	TBX2	p53kd	TBX2	p53kd	TBX2
>3 reduced	0.8	0.3	15.6	7.2	20.5	22.5
2-3x reduced	1.8	1.1	13.4	6.4	7.6	5.6
1.33-2x reduced	7.0	6.8	28.3	20.7	4.0	3.1
Equally expressed	26.4	28.8	42.3	65.2	1.6	2.3
Induced (>0.66x)	4.8	3.7	0.4	0.5	0.1	0.1
not expressed	59.2	59.3	not present	not present	not present	not present

Supplementary Table 1. Enrichment for shRNA vectors targeting suppressors of anchorless proliferation in LEGO libraries. The percentage of genome wide protein coding transcripts that are reduced to various extends in anchorless proliferating transformed DKO RASV12 TBX2 or p53kd cells compared to their arrested non-transformed DKO RASV12 counterparts is shown in the first two rows. The next two rows show the percentage of these reduced transcripts targeted by the LEGO-TBX2 and LEGO-p53kd libraries. The final two rows show the fold enrichment for these transcripts in the LEGO libraries.
