# Supplementary material for: RNAi screening of subtracted transcriptomes reveals tumor suppression by taurine-activated GABAA receptors involved in volume regulation
Source: PLoS One. 2018 May 22;13(5):e0196979. doi: 10.1371/journal.pone.0196979 (PMC5963783; doi:10.1371/journal.pone.0196979)
Supplement: S2 Table — Seventy-nine genes involved in suppression of anchorless proliferation are listed. Retesting of 15 selected genes using newly-designed shRNA vectors (marked *) supported their suppressive role. The suppressor transcripts they encode were ranked according to the score obtained by multiplying the absolute number of selected transforming vectors that targeted this transcript and the percentage this number represents of the maximal number of shRNA vectors potentially synthesized using each AluI site present in this transcript (Also see S1I Fig). In addition, its induction in DKO RASV12 MEFs after loss of anchoring, its repression by RASV12 expression, and its repression in anchorless DKO RASV12 TBX2 or DKO RASV12 p53kd cells compared to anchorless DKO RASV12 cells are shown. Genes reduced >1.66 fold in anchorless DKO RASV12 TBX2 or DKO RASV12 p53kd cells are shown in red, genes induced >1.66 fold in DKO RASV12 after loss of anchoring are shown in green; genes reduced >1.66 fold in attached DKO RASV12 compared to DKO cells are shown in magenta. They included 35 genes that were repressed (>1.66 fold) either in anchorless DKO RASV12 TBX2 cells (n = 28), in DKO RASV12 p53kd cells (n = 33), or in both fully transformed genotypes (n = 26). Note that 13 genes are regulated by anchoring, RASV12, p53 and TBX2. For each suppressor transcript, the accession number is shown and whether it encodes for a protein. Genes linked to taurine induced volume reduction are shown in red. For transcripts marked # Q-PCR was performed to validate expression data. Intriguingly, 4 transcripts encoding Cathepsins (A, B, D, L) were identified, suggesting these proteases play an important role in the suppression of anchorless growth. Furthermore two pairs of genes lying side by side in the genome were identified (Ctsa/Ptlp and Ptrf/Stat3). (PDF) [file pone.0196979.s002.pdf]

Gene	Ranking	No. vectors selected	*AluI* sites used (%)	Repression by RAS^{V12}	Repression by TBX2	Repression by p53	Induction anchorless	Type	Accession
Serpine2	1185	15	79	1.4	1.5	**2.8**	2.0	prot. coding	NM_009255
Ptp4a2*	1156	17	68	0.9	1.1	0.9	1.2	prot. coding	NM_008974
MK3#*	1088	14	78	0.9	**1.8**	**2.1**	2.0	prot. coding	NM_178907
Cd81	1071	17	63	1.1	1.1	1.3	1.3	prot. coding	NM_133655
2900097C17Rik*	816	17	48	nd	nd	nd	nd	non coding	NR_024329
Ctsd	806	13	62	1.2	1.4	1.2	1.6	prot. coding	NM_009983
AK087875*	770	10	77	nd	nd	nd	nd	non coding	AK087875
Pisd-ps3	736	16	46	1.4	**2.8**	**3.4**	2.9	non coding	NM_001004146
Sparc*	688	16	43	2.1	**2.5**	**5.0**	1.0	prot. coding	NM_009242
Ptrf*	630	21	30	0.7	**2.3**	1.3	0.5	prot. coding	NM_008986
Impact	585	13	45	2.0	1.5	1.6	1.7	prot. coding	NM_008378
Ctsb	561	17	33	1.4	1.0	1.1	1.8	prot. coding	NM_007798
Stk38l	540	20	27	2.0	1.4	**2.1**	2.7	prot. coding	NM_172734
Ywhae	528	11	48	0.7	0.7	0.8	0.7	prot. coding	NM_009536
Scpep1	507	13	39	0.9	1.0	1.1	1.0	prot. coding	NM_029023
Ephx1*	477	9	53	1.7	**3.2**	**2.7**	2.5	prot. coding	NM_010145
p21#*	477	9	53	1.0	**2.2**	**2.2**	2.6	prot. coding	NM_007669
Btg2	476	14	34	1.0	1.0	1.1	1.0	prot. coding	NM_007570
Rhobtb3	477	9	53	0.7	0.6	0.7	0.3	non coding	AK164957
Tmem59	451	11	41	1.5	1.2	1.2	1.2	prot. coding	NM_029565
Per3	402	6	67	nd	nd	nd	nd	non coding	AK012936
Cbr2*	400	4	100	4.5	**4.3**	**6.2**	2.6	prot. coding	NM_007621
ctsa*	396	12	33	3.7	**1.7**	**1.8**	3.3	prot. coding	NM_008906
Fbln2	375	15	25	0.6	**2.7**	**3.6**	2.0	prot. coding	NM_007992
Klh19	364	13	28	0.9	1.0	1.6	1.1	prot. coding	NM_172871
C730026J16	351	9	39	1.0	1.1	1.6	1.2	non coding	AK142305
**Tug1#***	352	16	22	1.9	1.4	**1.7**	1.4	non coding	NR_002321
Lamp2	340	10	34	4.2	**1.8**	**1.8**	4.2	prot. coding	NM_001017959
Stat3*	328	8	41	2.1	**2.0**	**2.0**	2.0	non coding	AK153170
Pbxip1*	296	8	37	3.2	**2.6**	**3.4**	2.6	prot. coding	AK047898
Rap2b	287	7	41	0.7	0.9	1.0	1.1	prot. coding	NM_028712
Canx	290	10	29	1.1	1.1	1.1	1.2	prot. coding	NM_007597
Bcl2l1	264	11	24	0.7	1.5	1.5	1.3	prot. coding	NM_009743
Trp53inp1*	253	11	23	2.5	**2.0**	**3.5**	3.9	prot. coding	NM_021897
Tsn	243	9	27	1.0	1.0	0.9	0.9	prot. coding	NM_011650
Ctsl	240	6	40	nd	nd	nd	nd	prot. coding	NM_009984
Ptp4a1	231	7	33	2.1	**1.7**	**2.8**	2.4	prot. coding	NM_011200
Cst3	225	5	45	2.2	**2.8**	**2.1**	1.9	prot. coding	NM_009976
KIAA0217	210	7	30	1.2	1.5	1.3	1.3	non coding	AK088176
Rab14	210	6	35	1.0	1.1	1.6	1.2	prot. coding	NM_026697
Tcf7l2	207	9	23	1.8	**2.1**	**1.9**	0.9	prot. coding	NM_009333
Rab31	208	8	26	1.3	1.0	1.2	2.0	prot. coding	NM_133685
Nras	209	11	19	0.8	0.8	1.2	1.0	prot. coding	NM_010937
**Actr2***	203	7	29	1.1	0.8	1.0	1.0	prot. coding	NM_146243
Ube2h	198	9	22	1.9	1.3	**1.8**	2.1	non coding	AK039198
Nqo1	196	7	28	0.7	1.1	1.0	1.1	prot. coding	NM_008706
Atp6v0d1	196	7	28	3.5	**1.8**	**2.5**	3.6	prot. coding	NM_013477
Sepw1	190	5	38	3.4	1.4	0.7	2.6	prot. coding	NM_009156
Idb3	192	6	32	1.0	1.1	**2.4**	0.6	prot. coding	NM_008321
Hspa5	192	6	32	0.9	0.9	0.9	0.8	prot. coding	NM_022310
mxd4*	180	5	36	1.9	**1.9**	**3.6**	1.5	non coding	AK189470
Cln2	182	7	26	2.7	**2.4**	**3.4**	3.8	prot. coding	NM_009906
**Ado#***	184	8	23	0.7	1.3	0.8	1.1	prot. coding	NM_001005419
**Nudt3**	180	3	60	1.0	1.0	0.9	1.0	non coding	AK003601
C330006A16Rik	176	4	44	0.8	1.0	1.3	1.0	non coding	AK077073
**Lrrc8a**	176	4	44	0.9	0.8	0.7	0.9	non coding	AK078148
**Gabarap**	176	4	44	1.9	**1.8**	1.3	1.9	prot. coding	NM_019749
Mir34a#*	176	8	22	2.0	**2.2**	**2.1**	2.1	microRNA	hsa-mir-34a
Dpyd	171	9	19	1.2	**2.3**	**3.2**	3.6	prot. coding	NM_170778
Sox4	174	6	29	1.8	1.2	1.3	1.8	non coding	AK054386
Ecm1	174	6	29	0.2	1.5	**2.0**	1.0	prot. coding	NM_007899
Tceb3	165	5	33	1.5	1.4	1.3	1.2	non coding	AK042376
Tnfaip1	168	8	21	0.7	0.8	1.2	0.8	prot. coding	NM_009395
Mxra8	161	7	23	3.8	**2.3**	**2.5**	2.5	prot. coding	NM_024263
Anxa8*	156	6	26	0.6	**4.8**	**5.2**	2.9	prot. coding	NM_013473
**GabrB3#***	156	6	26	3.7	**2.1**	**3.5**	4.3	prot. coding	NM_001038701
Vim	156	6	26	0.5	0.7	0.9	0.7	prot. coding	NM_011701
Qscn6	147	7	21	1.0	**1.8**	**1.8**	1.7	non coding	AK171601
Pcnp	145	5	29	1.0	1.0	1.4	1.4	prot. coding	AK041055
Akr1a4	145	5	29	1.2	1.5	1.1	1.5	prot. coding	NM_021473
Golt1b	145	5	29	1.0	0.9	0.9	0.9	prot. coding	NM_025872
**Arpc1b**	145	5	29	0.8	1.1	0.9	1.0	prot. coding	NM_023142
AK088773	144	4	36	nd	nd	nd	nd	prot. coding	AK088773
Pltp	144	6	24	2.0	**3.5**	**11.9**	7.1	prot. coding	NM_011125
Cd44	132	6	22	0.6	0.7	1.0	1.1	non coding	AK045226
E130309L16Rik	132	6	22	0.9	1.5	**1.7**	0.9	prot. coding	AK053808
Zfp57	132	6	22	0.7	0.8	0.8	0.6	prot. coding	NM_009559
**Moesin**	132	6	22	0.3	0.8	0.9	0.6	prot. coding	NM_010833
Ece1	133	7	19	2.9	**3.2**	**2.8**	2.6	prot. coding	NM_199307

Supplementary Table 2. Suppressors of anchorless proliferation. Seventy-nine genes involved in suppression of anchorless proliferation are listed. Retesting of 15 selected genes using newly-designed shRNA vectors (marked *) supported their suppressive role. The suppressor transcripts they encode were ranked according to the score obtained by multiplying the absolute number of selected transforming vectors that targeted this transcript and the percentage this number represents of the maximal number of shRNA vectors potentially synthesized using each *Alu*I site present in this transcript (Also see Supplementary Fig. 1I). In addition, its induction in DKO RAS^{V12} MEFs after loss of anchoring, its repression by RAS^{V12} expression, and its repression in anchorless DKO RAS^{V12} TBX2 or DKO RAS^{V12} p53kd cells compared to anchorless DKO RAS^{V12} cells are shown. Genes reduced >1.66 fold in anchorless DKO RAS^{V12} TBX2 or DKO RAS^{V12} p53kd cells are shown in red, genes induced >1.66 fold in DKO RAS^{V12} after loss of anchoring are shown in green; genes reduced >1.66 fold in attached DKO RAS^{V12} compared to DKO cells are shown in magenta. They included 35 genes that were repressed (>1.66 fold) either in anchorless DKO RAS^{V12} TBX2 cells (n=28), in DKO RAS^{V12} p53kd cells (n=33), or in both fully transformed genotypes (n=26). Note that 13 genes are regulated by anchoring, RAS^{V12}, p53 and TBX2. For each suppressor transcript, the accession number is shown and whether it encodes for a protein. Genes linked to taurine induced volume reduction are shown in red. For transcripts marked # Q-PCR was performed to validate expression data. Intriguingly, 4 transcripts encoding Cathepsins (A, B, D, L) were identified, suggesting these proteases play an important role in the suppression of anchorless growth. Furthermore two pairs of genes lying side by side in the genome were identified (*Ctsa*/*Ptlp* and *Ptrf*/*Stat3*).
