# Supplementary material for: RNAi screening of subtracted transcriptomes reveals tumor suppression by taurine-activated GABAA receptors involved in volume regulation
Source: PLoS One. 2018 May 22;13(5):e0196979. doi: 10.1371/journal.pone.0196979 (PMC5963783; doi:10.1371/journal.pone.0196979)
Supplement: S1 Fig — A. Schematic representation of adaptors and primers used for subtractive hybridization. For the production of LEGO libraries, we designed a set of oligonucleotide adapters A, B, and C that allow (1) PCR-based suppression subtractive hybridization (SSH) (adapted from ref. 7), and (2) enzymatic processing of selected fragments into shRNA vectors. For SSH, adapters A and B contain two priming sites for a first round of PCR (dark blue) and nested PCR (light blue and yellow). Adaptor A and B lack a 5' phosphate group to prevent ligation of the antisense adaptor strand to the cDNA. In addition, part of adapter A participates in library processing (red) using recognition sites for MmeI (violet), BpmI (orange) and NbBCIA (green). The phosphorylated adapter C has a degenerate 2 nt overhang, contains the 9 nt sequence destined to form the loop of the shRNA molecules (purple), and forms two BsgI sites (pink) upon synthesis of the complementary strand. The adapter for pRETRO Super modification is inserted into pRETRO Super behind the H1 promotor using BglII and HindIII sites. Subsequent BsgI (pink) digestion of the adapted pRETRO Super creates two CT overhangs (red): one that follows the transcription initiation signal of the H1 promotor (brown; CCCC) and the other in front of the transcription termination signal (dark green; TTTTT). This allows insertion of the shRNA inserts produced by our protocol, which have AG overhangs after BpmI digestion. These overhangs are determined by the AluI digestion that precedes adaptor A ligation: the cDNA ligated to the adapter A has blunt CT ends as a result of the AluI site (= AGCT), which are transformed in the AG overhangs as BpmI cuts exactly here during digestion of SSH PCR product (also see S1I Fig; step 7). The G in front of the CT overhang (pale yellow) reconstitutes part of this AluI restriction site, and causes the transcribed shRNA molecules to start with GCT. B. Sequences of the adapters and primers used for subtractive hybridiza [file pone.0196979.s003.pdf]

Supplementary Figure 1. Optimization of subtractive hybridization.

A. Schematic representation of adaptors and primers used for subtractive hybridization. For the production of LEGO libraries, we designed a set of oligonucleotide adapters A, B, and C that support (1) PCR-based suppression subtractive hybridization (SSH) (adapted from ref. 7), and (2) enzymatic processing of selected fragments into shRNA vectors. For SHH, adapters A and B contain two priming sites for nested PCR (light blue, dark blue and yellow). Adaptor A and B lack a 5' phosphate group to prevent ligation of the antisense adaptor strand to the cDNA. In addition, part of adapter A participates in library processing (red) using recognition sites for *Mme*I (violet), *Bpm*I (orange) and N*bBC*IA (green). The phosphorylated adapter C has a degenerate 2 nt overhang, contains the 9 nt sequence destined to form the loop of the shRNA molecules (purple), and forms two *Bsg*I sites (pink) upon synthesis of the complementary strand. The adapter for pRETRO Super modification is inserted into pRETRO Super behind the H1 promotor using *Bgl*II and *Hind*III sites. Subsequent *Bsg*I (pink) digestion of the adapted pRETRO Super creates two CT overhangs (red): one that follows the transcription initiation signal of the H1 promotor (brown; CCCC) and the other in front of the transcription termination signal (dark green; TTTTT). This allows insertion of the shRNA inserts produced by our protocol, which have AG overhangs after *Bpm*I digestion. These overhangs are determined by the *Alu*I digestion that precedes adaptor A ligation: the cDNA ligated to the adapter A has blunt CT ends as a result of the *Alu*I site (= AGCT), which are transformed in the AG overhangs as *Bpm*I cuts exactly here during digestion of SHH PCR product (also see Supplementary Fig1I; step 7). The G in front of the CT overhang (pale yellow) reconstitutes part of this *Alu*I restriction site, and causes the transcribed shRNA molecules to start with GCT.

B. Sequences of the adapters and primers used for subtractive hybridization. Note that the first PCR uses only one primer recognizing a priming site present on both Adapter A and B (dark blue). Klenow Primer 3 serves as a primer for Klenow polymerase. Primers used for deep sequencing of shRNA inserts can be given an 8 nt barcode (green) and sequenced all together. Attb1 and attb2 recombination sites required for gateway cloning are shown in red.

C. Schematic representation of PCR-based SHH used to select sequences for LEGO library production. The subtraction and shRNA production procedure was optimized using a pair of isogenic embryonic stem cell lines, H- (called driver cDNA) and H+ (called tester cDNA), the latter expressing the Hygromycin B resistance gene hyg. (1) Polyadenylated RNA isolated from tester (H+; red) and driver (H-; light blue) cells is converted to cDNA and digested with *Alu*I. 5’ ends (not 3’ ends) of *Alu*I-digested tester cDNA are ligated to either Adapter A (1a) or B (1b) in separate reactions. (2) The Adapter A- and B-ligated tester fragments are hybridized separately to an excess of *Alu*I-digested driver cDNA. As an adaptation to the original protocol (Diatchenko et al., 1996), we prolonged this this first hybridization step until equilibrium was reached. Due to the second order kinetics of hybridization, which causes the more abundant fragments to re-anneal faster, an equal number of each of the AluI fragments is expected to remain single stranded at equilibrium (also see Supplementary Fig. 1D). Moreover, the ratio of adaptor-ligated to adaptor-less single-strands at equilibrium depends on the relative expression levels in the tester and the driver cells: more single strands will carry an adapter if the corresponding RNA was more abundant in the tester cells than in the driver cells, and visa versa.

(3) Subsequently, the Adapter A- and B-containing samples were mixed, and further hybridization of remaining single strands was enforced by adding the volume exclusion agent polyethylene glycol (PEG). In this step,

hybrids carrying both the Adapter A and B at their ends can form. Again, their abundance depends on the relative expression levels in the tester and the driver cells.
(4) After filling in the complementary adapter strand, these hybrids can be exponentially amplified by nested PCR. Amplification of hybrids carrying two identical adapters is suppressed by intrastrand folding (to prevent intrastrand folding, the sites required for subsequent enzymatic processing of LEGO libraries are only present on adaptor A and not on adaptor B). The PCR product will be enriched for fragments that were overrepresented in the tester cell line H+ and contain normalized and reduced numbers of equally and less expressed fragments, respectively.
D. Schematic overview of the kinetics of re-annealing during subtractive hybridization. After mixing the driver and tester cDNA fragments, the strands are melted and allowed to re-anneal. Adapter A and B ligated fragments are hybridized to the driver cDNA in separate reactions. At the start of re-annealing, the different single-stranded *Alu*I fragments are present at different concentrations because of different expression levels in the cells. Re-annealing follows second order kinetics. This means that more abundant cDNA fragments, which meet more often, re-anneal faster than scarce cDNA fragments, until an equilibrium concentration is reached (colored lines). At equilibrium, a normalized number of each different *Alu*I fragment present in the mixture is expected to remain single stranded (black line). After the first hybridization has reached equilibrium, adapter A and B ligated samples are mixed. To stimulate further re-annealing, the volume exclusion agent PEG is added to cause molecular crowding increasing the effective concentration.
E. Quantification using spotblots. To determine the success of subtractive hybridization, the abundance of a panel of 9 genes was determined in the *Alu*I digested tester cDNA and the subtracted library. Library and cDNA were radiolabelled and hybridized to a nitrocellulose filter on which the differentially expressed *hyg* gene, the abundant *actin*, *tubulin*, and *E cadherin* genes and the lower expressed *Msh6*, *p53*, *CDK1* and *Cyclin A* genes were spotted. In tester cDNA, *actin, tubulin* and *E cadherin* transcripts were abundant, while *Msh6*, *p53, CDK*1, *Rb, Cyclin* A and *hyg* transcripts were scarce. In de subtracted cDNA library, all fragments were normalized to low equal levels, while the *hyg* fragments were strongly enriched.
F. Optimization of enrichment by subtractive hybridization: hybridization time. Normalization and enrichment improved by prolonged duration of the first hybridization. We first determined the time required to reach equilibrium for optimal normalization during the first hybridization and examined the effect of PEG addition. Adapter A and B-ligated tester H+ cDNA preparations were separately mixed with a fixed amount of driver H- cDNA at (ratio of 1:35) and the first hybridization was allowed to proceed from 0 to 45 hours in the presence of 5% PEG. Subsequently, the Adapter A- and B-containing samples were mixed and the PEG concentration was raised to 15%. After the second hybridization period (24 h), the subtracted libraries were amplified by PCR and the abundance of a panel of 9 gene sequences was determined. To measure the abundance of different genes in the subtracted libraries, PCR products were radiolabelled and hybridized to a nitrocellulose filter on which a panel of 9 genes was spotted. After 45 hours, the abundant *actin*, *tubulin*, *E cadherin* and *Msh6* genes and the lower expressed *p53*, *CDK1, Rb* and *Cyclin A* genes, which are equally expressed in the two cell lines H+ and H-, were reduced to normalized low levels in the subtracted library, but not completely removed. In contrast, *hyg* sequences, which are specific to the tester cDNA, were strongly enriched.

G. Optimization of enrichment by subtractive hybridization: ratio tester/driver. Subsequently, we determined the effect of the amount of driver cDNA added to the first hybridization on the efficacy of subtraction. The procedure was followed using increasing amounts of driver H- cDNA during the first hybridization. As expected, normalization and enrichment improved when increasing amounts of driver cDNA were added to the reaction.

H. Optimization of enrichment by subtractive hybridization: polyethyleneglycol. The addition of PEG during the second hybridization was essential for optimal subtractive hybridization. Adapter A- and B-ligated tester H+ cDNA preparations were separately mixed with a fixed amount of driver H- cDNA at (ratio of 1:35) and the first hybridization was allowed to proceed from 0 to 45 hours in the presence of 5% PEG. Subsequently, the Adapter A- and B-containing samples were mixed with or without raising the PEG concentration to 15%. After the second hybridization period (24 h), the subtracted libraries were amplified by PCR and the relative abundance of *hyg* was determined. Without addition of PEG, subtractive hybridization was less effective and no subtracted libraries could be amplified when the first hybridization period proceeded more than 18 hours.

In conclusion: We found the efficacy of subtractive hybridization to be highest by adding 60-fold excess of driver cDNA to the first hybridization reaction and by allowing this step to proceed for 45 h (Supplementary Fig. 2F,G). Importantly, the addition of PEG during the second hybridization step was crucial for optimal subtractive hybridization: it increased enrichment of *hyg* sequences more than 12 fold (Supplementary Fig. 2D).

I. Enzymatic production of shRNA vectors from subtracted libraries. Using restriction sites located on the adaptor A flanking the SHH PCR product (the subtracted cDNA library), and on adaptor C, the selected cDNA sequences can be processed into inverted repeats and inserted into pRETRO Super vectors to produce the subtracted retroviral LEGO shRNA library. Because adaptor A is ligated to both ends of each *Alu*I fragment, two shRNA vectors can result from each *Alu*I site. Note that the cDNA parts have AG ends resulting from *Alu*I digestion before adaptor ligation. (1) Digestion with *Mme*I (violet), which cuts outside its recognition site, leaves 18 base pairs of cDNA (blue) attached to Adapter A. (2) Using its 2 nt degenerate overhang (N_1N_2), the looped Adapter C carrying the 9 nt shRNA loop is ligated to the cDNA, forming a short hairpin. (3) After inactivating the ligase, *N.Bbvc* IA (green) nicked Adapter A. (4) The botinylated primer Nested PCR2a used to amplify the subtracted library allows isolation of the nicked hairpins using streptavidin-coated beads. (5) Subsequent heating inactivated *N.Bbvc* IA and, due to the nick, the hairpins were released from the bead, allowing primer annealing to the now exposed single-stranded region. (6) Primer extension by Klenow DNA polymerase generated double-stranded inverted repeat sequences. (7) *Bpm*I (orange), which cuts 16 bp away from its recognition site, was used to remove the duplicated adapter A ends, releasing inverted repeats with AG overhangs. (8) These are isolated after separation on gel, and (9) ligated behind the H1 promoter (brown) of a modified pRETRO Super backbone with CT overhangs (Supplementary Fig. 1). (10) Finally, part of Adapter C is removed using *Bsg*I (pink), which cuts 16 bp from its restriction site, creating functional pRetro SUPER LEGO shRNA vectors.

J. Functionality of shRNA vectors. Using our procedure, we generated eleven different vectors from the 998 bp *hyg* open reading frame. To confirm their functionality, the knockdown level they achieved was measured by quantitative PCR. After infection of *hyg*+ cells, 9 vectors reduced *hyg* transcripts by 40 to 80 percent.

A.

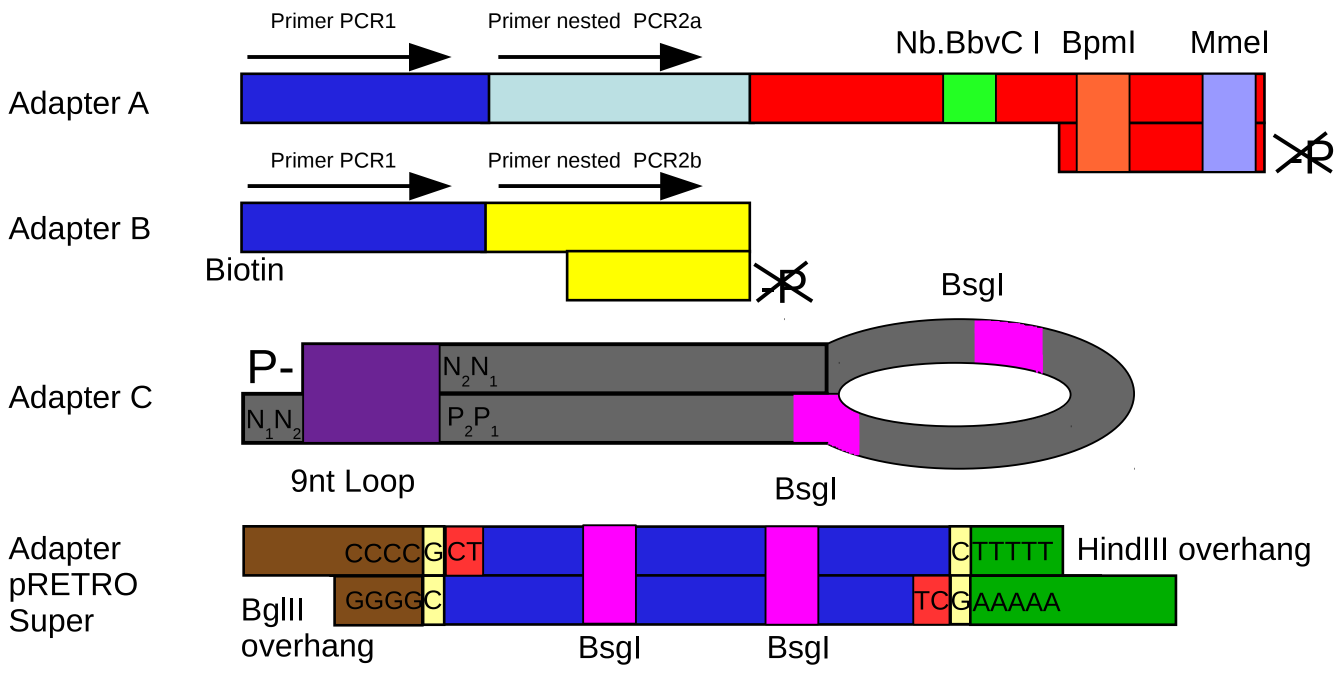

B. **Adapter A**

GTATTACCGCACTCACTTGGAGCATCGTCCTGGCGTCTGGCTGAGGGTAGCTGGAGAGGCGATCCGACCG

Antisense Adaptor A (lacks phosphate for ligation)

CGGTCGGATCGC

Adapter B

BiotinAAAAAAAAAAAAAAAAAAAAGTATTACCGCACTCACTTGGACTTCTGTCACCGTCACCGCATAGCTCATCTACGTCTTCC

Antisense Adaptor B (lacks phosphate for ligation)

GGAAGACGTAGATGAGC

Primer PCR1

GTATTACCGCACTCACTTGGA

Primer Nested PCR 2a

BiotinCATCGTCCTGGCGTCTGGCT

Primer Nested PCR 2b

CTTCTGTCACCGTCACCGCATAG

Klenow Primer 3

CATCGTCCTGGCGTCTGGC

Looped Adaptor C

TTCAAGAGAN_2N_1GCGTTGCACCGGTGCTGCACCGGTGCAGCGCP_1P_2TCTCTTGAAN_2N_1

(N can be A, G, C, or T; P_x pairs with N_x)

Adapter for pRetroSuper modification prior to insertion of hairpins (sense)

GATCCCCGCTGCCGTGCAGTTAACCTGCACCCGAGCTTTTTGGAAA

Adapter for pRetroSuper modification prior to insertion of hairpins (antisense)

AGCTTTTCCAAAAAGCTCGGGTGCAGGTTAACTGCACGGCAGCGGG

Amplification hairpin and addition experiment tag for Deep sequencing

CAAGCAGAAGACGGCATACGAGCTCNNNNNNNNTCCTCCCTTTATCCAGCCCTCACTC

AATGATACGGCGACCACCGAGATCTTACCACCTATGTATGAGACCACAGATCCCCGC

Sequence primer shRNAi hairpin

TACCACCTATGTATGAGACCACAGATCCCCGCT

Sequence primer tag

TAGAGAAGGAGTGAGGGCTGGATAAAGGGAGGA

Amplification shRNAi hairpin + H1 promotor and addition flanking gateway recombination sites

GGGGACAAGTTTGTACAAAAAAGCAGGCTCAACCCGCTCCAAGGAATCG

GGGGACCACTTTGTACAAGAAAGCTGGGTAGTCTTTTCACCTGGCCCGCAT

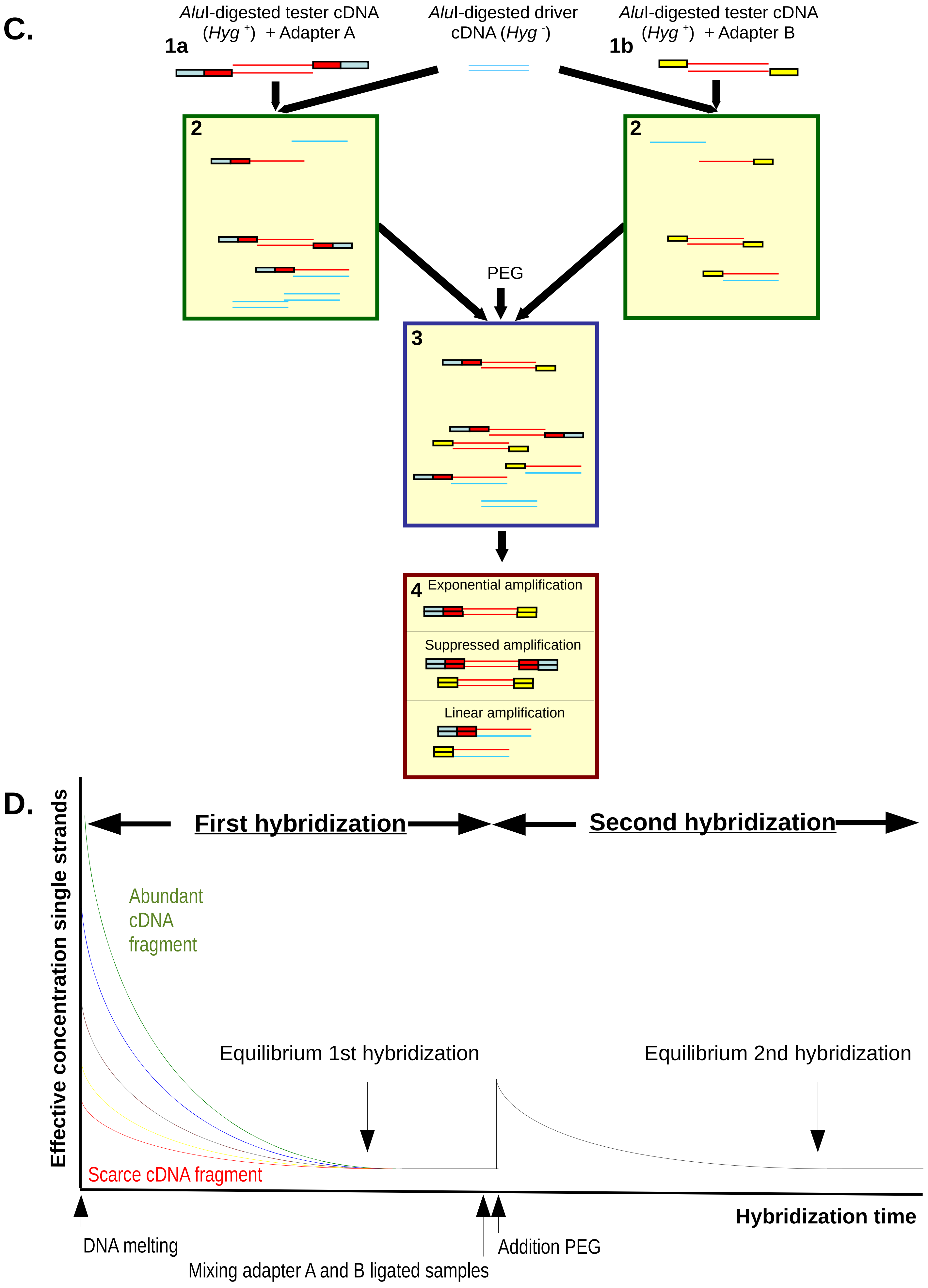

C.
AluI-digested tester cDNA (Hyg +) + Adapter A
1a
AluI-digested driver cDNA (Hyg -)
AluI-digested tester cDNA (Hyg +) + Adapter B
1b
2
2
PEG
3
4
Exponential amplification
Suppressed amplification
Linear amplification
D.
Effective concentration single strands
First hybridization
Second hybridization
Abundant cDNA fragment
Equilibrium 1st hybridization
Equilibrium 2nd hybridization
Scarce cDNA fragment
Hybridization time
DNA melting
Addition PEG
Mixing adapter A and B ligated samples

C.

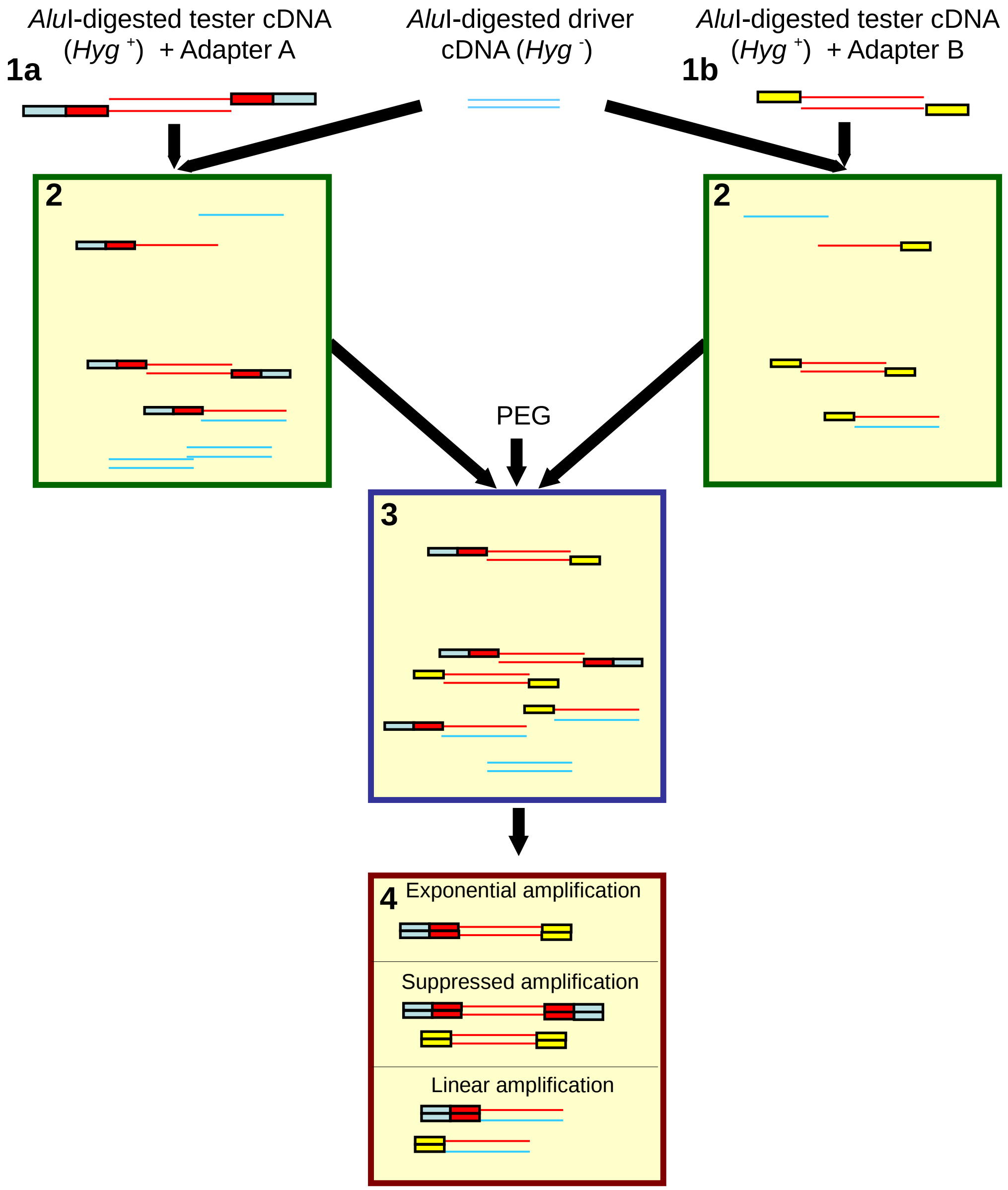
*Alu*I-digested tester cDNA
(*Hyg* +) + Adapter A
1a
*Alu*I-digested driver
cDNA (*Hyg* -)
*Alu*I-digested tester cDNA
(*Hyg* +) + Adapter B
1b
2
2
PEG
3
4
Exponential amplification
Suppressed amplification
Linear amplification

D.

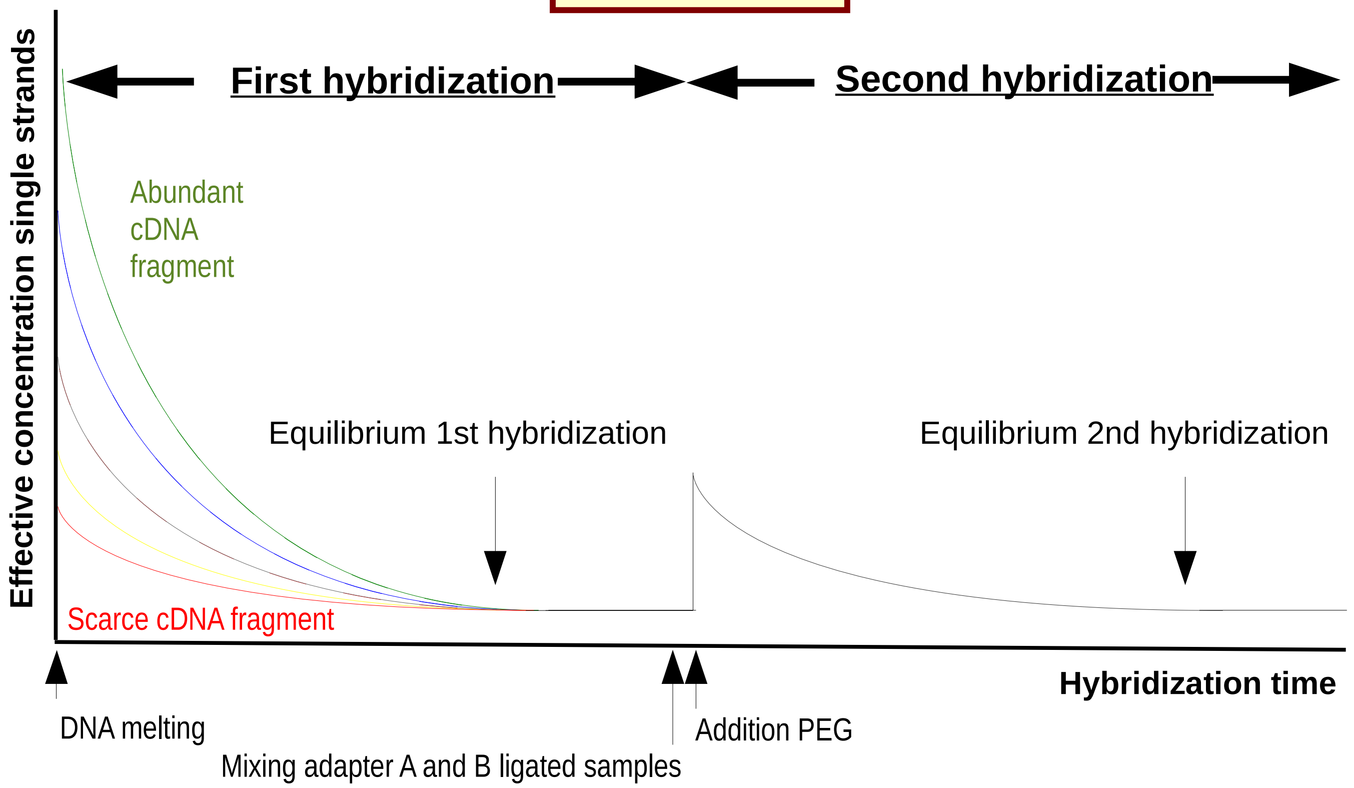
Effective concentration single strands
First hybridization
Second hybridization
Abundant
cDNA
fragment
Equilibrium 1st hybridization
Equilibrium 2nd hybridization
Scarce cDNA fragment
Hybridization time
DNA melting
Addition PEG
Mixing adapter A and B ligated samples

E

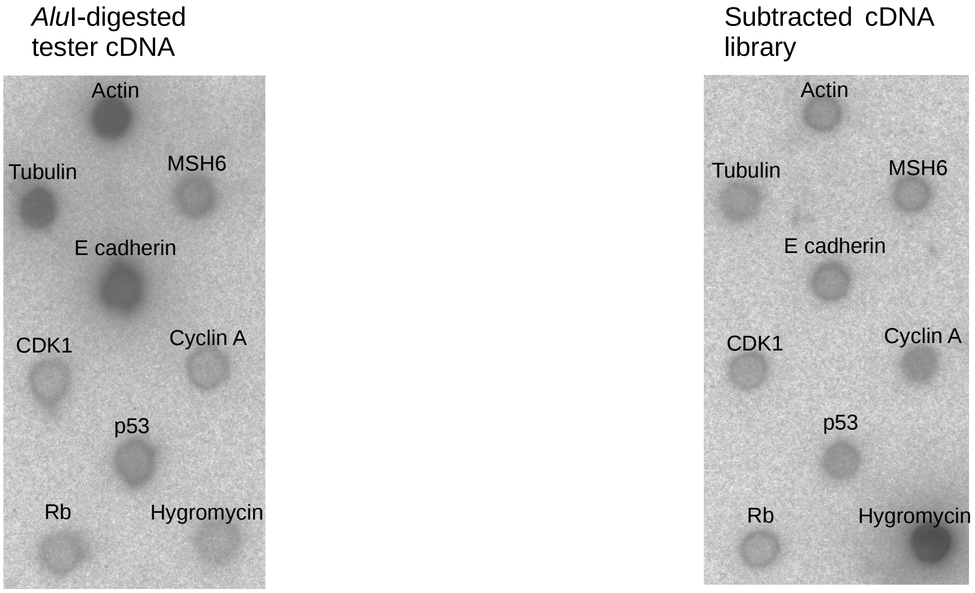
AluI-digested tester cDNA
Actin
Tubulin
MSH6
E cadherin
CDK1
Cyclin A
p53
Rb
Hygromycin
Subtracted cDNA library
Actin
Tubulin
MSH6
E cadherin
CDK1
Cyclin A
p53
Rb
Hygromycin

F

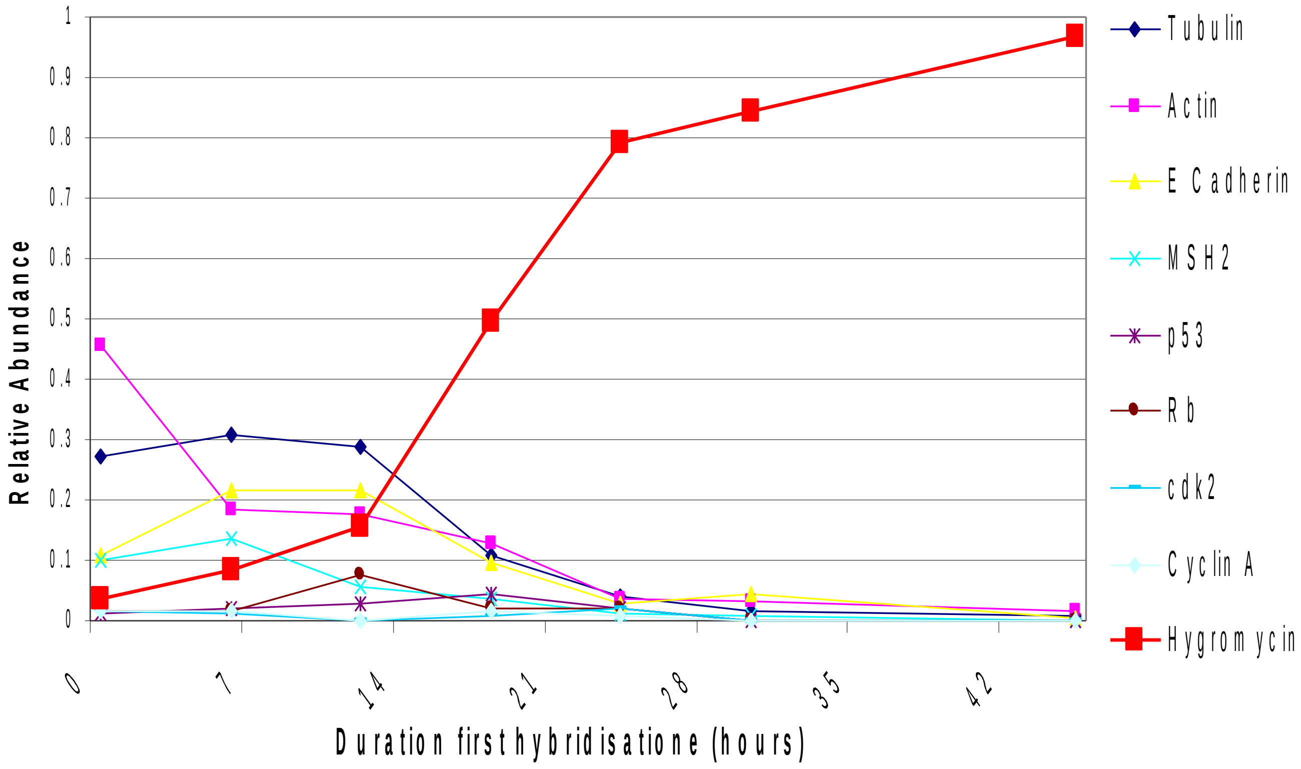
Relative Abundance
1
0.9
0.8
0.7
0.6
0.5
0.4
0.3
0.2
0.1
0
0
7
14
21
28
35
42
Duration first hybridisatione (hours)
Tubulin
Actin
E Cadherin
MSH2
p53
Rb
cdk2
Cyclin A
Hygromycin

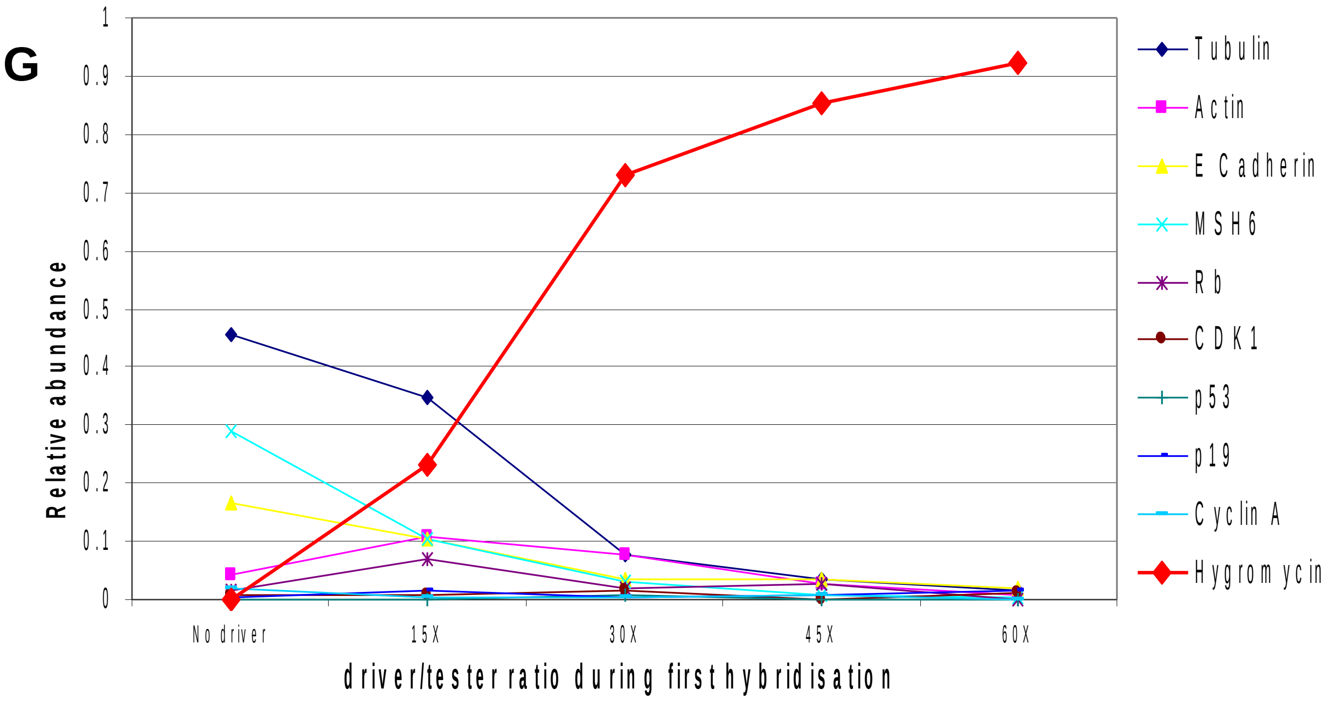
G
Relative abundance
driver/tester ratio during first hybridisation
No driver
15X
30X
45X
60X
Tubulin
Actin
E Cadherin
MSH6
Rb
CDK1
p53
p19
Cyclin A
Hygromycin

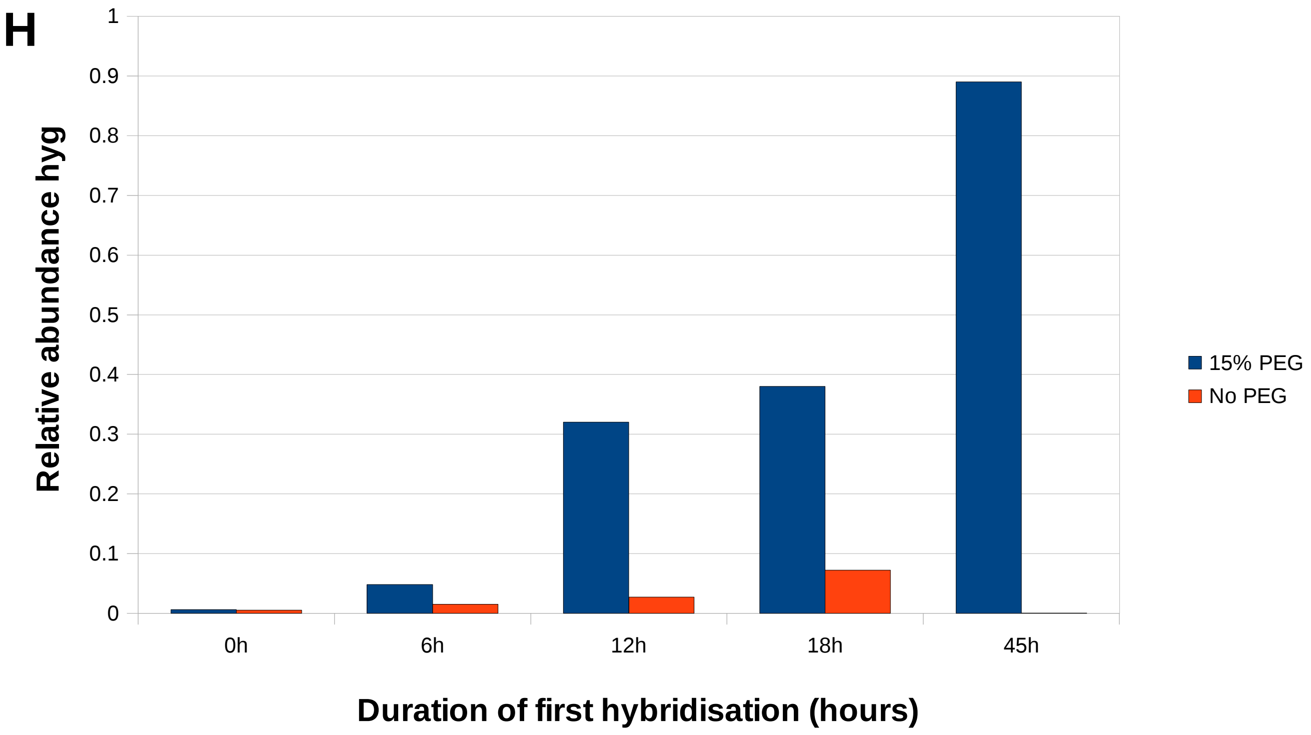
H
Relative abundance hyg
Duration of first hybridisation (hours)
0h
6h
12h
18h
45h
15% PEG
No PEG

I

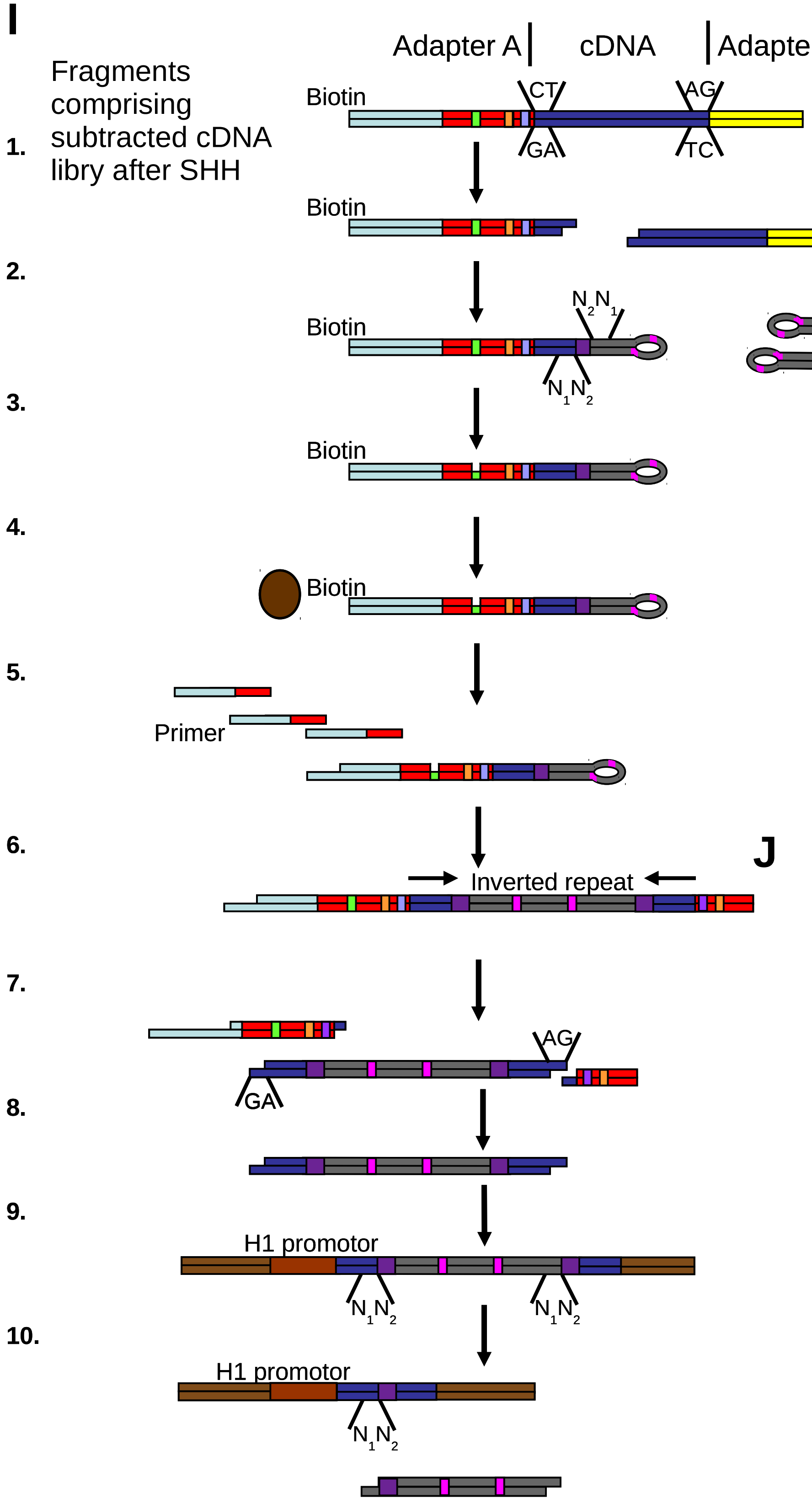
Adapter A
cDNA
Adapter B
Adapter C
Fragments comprising subtracted cDNA libry after SHH
1.
Biotin
CT
GA
AG
TC
N2N1
N1N2
Biotin
2.
Biotin
N2N1
N1N2
3.
Biotin
4.
Biotin
5.
Primer
6.
Inverted repeat
7.
AG
GA
8.
9.
H1 promotor
N1N2
N1N2
10.
H1 promotor
N1N2

J

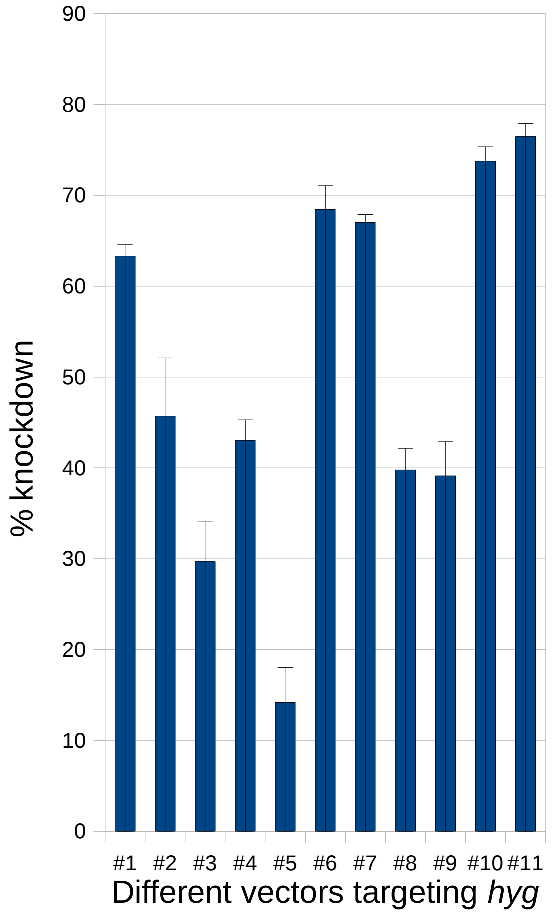
% knockdown
0
10
20
30
40
50
60
70
80
90
#1
#2
#3
#4
#5
#6
#7
#8
#9
#10
#11
Different vectors targeting *hyg*
