# Supplementary material for: RNAi screening of subtracted transcriptomes reveals tumor suppression by taurine-activated GABAA receptors involved in volume regulation
Source: PLoS One. 2018 May 22;13(5):e0196979. doi: 10.1371/journal.pone.0196979 (PMC5963783; doi:10.1371/journal.pone.0196979)

Supplementary Figure 2. Validation of various selected hits as suppressors of anchorless proliferation.

A. Newly designed shRNA sequences inserted into pRETRO Super vectors used for knockdown of identified suppressor transcripts.
B. Level of knockdown achieved for a subset of these shRNA vectors in typical experiments as measured by quantitative PCR is depicted.
C. Retesting of 17 selected hits. Newly designed shRNA vectors targeting selected suppressor transcripts were tested for their effect on proliferation in colony forming assays in methylcellulose. Pictures of wells were taken 3 weeks after $5x10_4$ cells were seeded per well.
D. miR-34a is a suppressor of anchorless proliferation. Colony forming assays showing increased proliferation of *Rb*$_{-/-}$*107*$_{-/-}$ RAS$_{V12}$ MEFs in methylcellulose upon knockdown (kd) of the non-coding suppressor transcript miR-34a. Viral co-expression (expr) of miR-34a in *Rb*$_{-/-}$*107*$_{-/-}$ RAS$_{V12}$ TBX2 MEFs largely reverted transformation. The Luc vector (Mock) was used as a negative control for miR-34a. Pictures of wells were taken 3 weeks after $5x10_4$ cells were seeded per well.
E. MK3 is a suppressor of anchorless proliferation. Colony forming assays showing increased proliferation of *Rb*$_{-/-}$*107*$_{-/-}$ RAS$_{V12}$ MEFs in methylcellulose upon knockdown (kd) of the p38 effector kinase MK3. Viral coexpression (expr) of MK3 in *Rb*$_{-/-}$*107*$_{-/-}$ RAS$_{V12}$ TBX2 MEFs largely reverted transformation. The Luc vector (Mock) was used as a negative control for MK3. Pictures of wells were taken 3 weeks after $5x10_4$ cells were seeded per well.
F. Network analysis of hits. Network revealed by Ingenuity analysis using 23 hits that were induced (1.66-7.05 fold) in non-transformed DKO RAS$_{V12}$ cells after loss of anchoring, but repressed (1.66-11.9 fold) in both transformed genotypes during anchorless proliferation.
G. GABA receptors and taurin are involved in suppression of anchorless proliferation. Newly designed shRNA vectors targeting GABA receptors and ADO were tested for their effect on proliferation in colony forming assays in methylcellulose. Pictures of wells were taken 3 weeks after $5x10_4$ cells were seeded per well.

A. Short hairpin sequences in pRetro Super

Gene	Sort hairpin sequence in pRETRO Super
2900097C17Rik	GACACCACTTTGTTAACGATGATTTCCAGTGTTGAGAGGAAATCATCGTTAACAAAGTGGTGTC
Actr2	GCAGCCGACATTGATACCAGACCAGTGTTGAGAGGTCTGGTATCAATGTCGGCTGC
ADO	GGACCCTAAAGCGCTTCCATCCTAACCAGTGTTGAGAGGTTAGGATGGAAGCGCTTTAGGGTCC
AK087875	CAGGATCACTCTACGTGGGTCACATCCAGTGTTGAGAGGATGTGACCCACGTAGAGTGATCCTG
Anxa8	GGAACCAATGAGCAGGCCATCATACCAGTGTTGAGAGGTATGATGGCCTGCTCATTGGTTCC
Cbr2	CAGATGGTAGCCAGGGACATGATTATTGATATCCGTAATCATGTCCCTGGCTACCATCTG
Ctsa	GAGGACACACTTGTAGTCCAGGATTCCTCTCAACACTGGAATCCTGGACTACAAGTGTGTCCTC
Ephx1	CGCTTCCACTATGGCTTCAACTCCATTCAAGAGATGGAGTTGAAGCCATAGTGGAAGCG
GabrA5	GCCAACTAGTTCTGTACAAGACCTCTCAACACTGGTCTTGTACAGAACTAGTTGGC
GabrB3	GCTCCCACAGTTCTCCATTGTCCTCTCAACACTGGACAATGGAGAACTGTGGGAGC
GabrG3	AGACTCCAGTTTGTTGTGCTACCTTTCCAGTGTTGAGAGGAAAGGTAGCACAACAAACTGGAGTC
hspb1	CGGAGATCACCATTCCGGTTACTTTCCAGTGTTGAGAGGAAAGTAACCGGAATGGTGATCTCCG
map2k3	AGACAGCCTCTGGTTACCACCTTTACCAGTGTTGAGAGGTAAAGGTGGTAACCAGAGGCTGTCT
map2k3	GGATCAACCCTGAACTGAATCCCTCTCAACACTGGGATTCAGTTCAGGGTTGATCC
Map2k6	GCCTTCCCTAACGTTGCAACTCCAGTGTTGAGAGGAGTTGCAACGTTAGGGAAGGC
MK3	GCTAATCCGCCTGCTCCTGTCTCTTGAACAGGAGCAGGCGGATTAGC
MK3	GCTCTTTATCCCCAAGTTCTCTCTTGAAGAACTTGGGGATAAAGAGC
MK3	GCTGCCTGCCTGCTTTTTCCTTCAAGAGAGGAAAAAGCAGGCAGGCAGC
Mir34a	GGCAACTGGTTTAGTAGCCTGACTACCAGTGTTGAGAGGTAGTCAGGCTACTAAACCAGTTGCC
mxd4	GCACAGGACTTTGTTTAAAGGCCTCTCAACACTGGCCTTTAAACAAAGTCCTGTGC
neg. Contr.	GACAGCCGCAGAGCGTTTGAGTATACCTCTCAACACTGGTATACTCAAACGCTCTGCGGCTGTC
Pbxip1	GAAAGATGGGAACTCCCATTCTGTACCAGTGTTGAGAGGTACAGAATGGGAGTTCCCATCTTTC
Ptp4a2	GCAGAAGTTGACCAGACAAAGCCAGTGTTGAGAGGCTTTGTCTGGTCAACTTCTGC
Ptrf	GCCGCAACTTCAAAGTCATGATCTACCTCTCAACACTGGTAGATCATGACTTTGAAGTTGCGGC
Sparc	GGACATCAACAAGGATCTGGTGATCCAGTGTTGAGAGGATCACCAGATCCTTGTTGATGTCC
stat3	GCAGCAGCTGAACAACATGCCTCTCAACACTGGCATGTTGTTCAGCTGCTGC
Trp53inp1	GAATGGATTCTTGTTGACTTCTAGCCAGTGTTGGAGGCTATGAAGCAACAAGAATCCATC
Tug1	GCTTCATTATTAATCTGCCTGTTCTCCTCTCAACACTGGAGAACAGGCAGATTAATAATGAAGC
P21	GCCCTCACTCTGTGTGTCTTTCAAGAGAAGACACACAGAGTGAGGGC

B. Levels of knockdown achieved by various pRETRO Super vectors

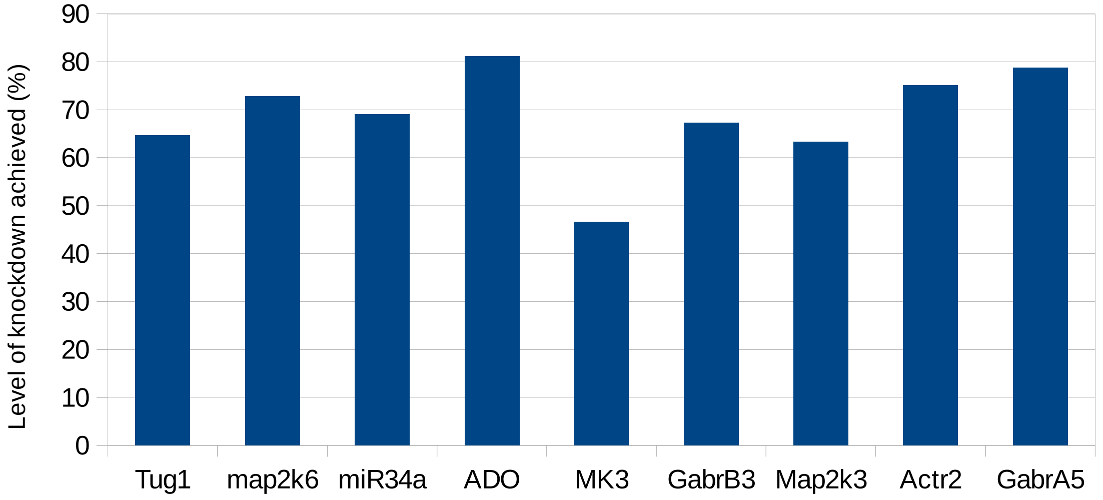

C. Colony forming assays of cells expressing knockdown vectors directed against various genes.

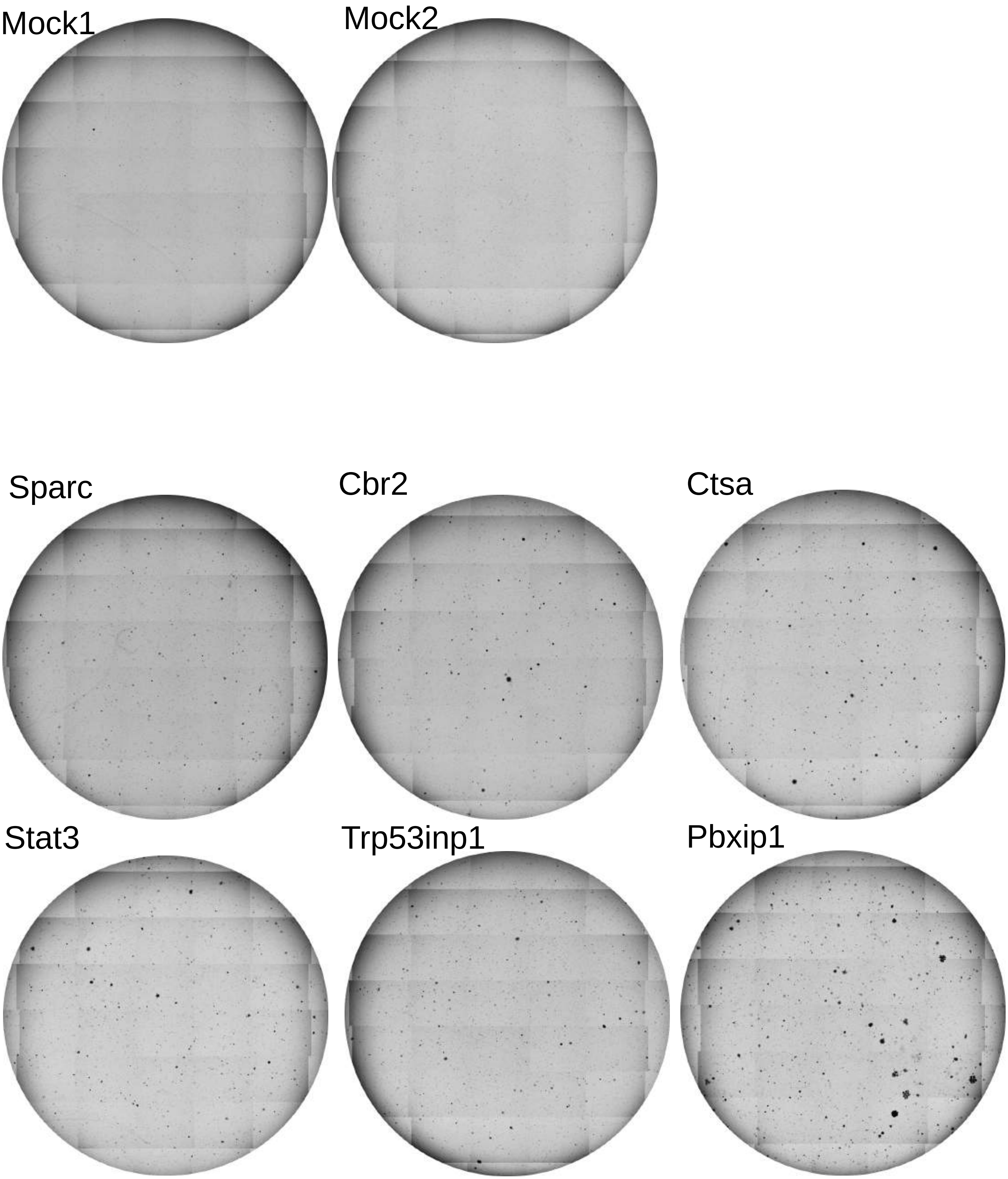

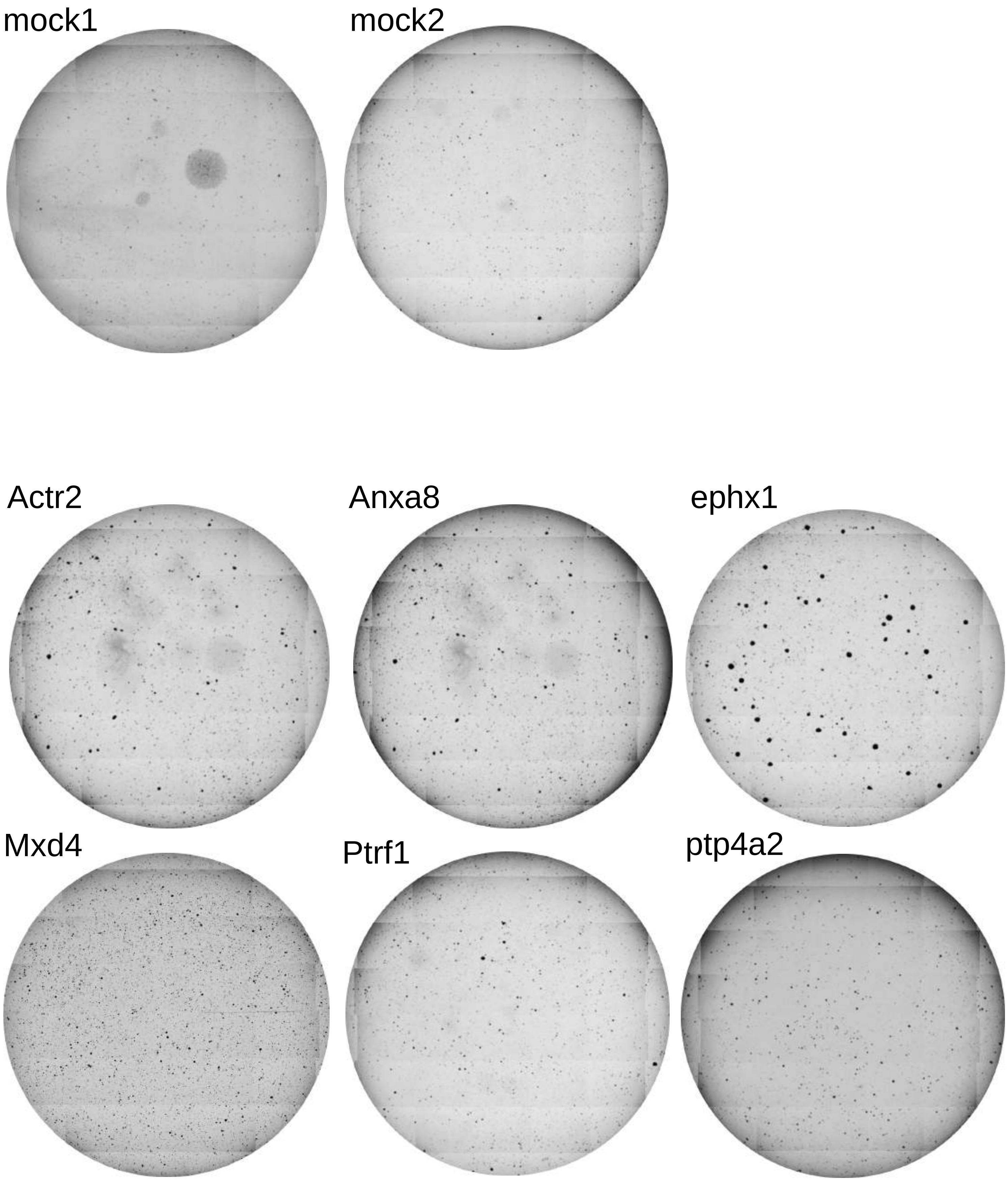
mock1
mock2
Actr2
Anxa8
ephx1
Mxd4
Ptrf1
ptp4a2

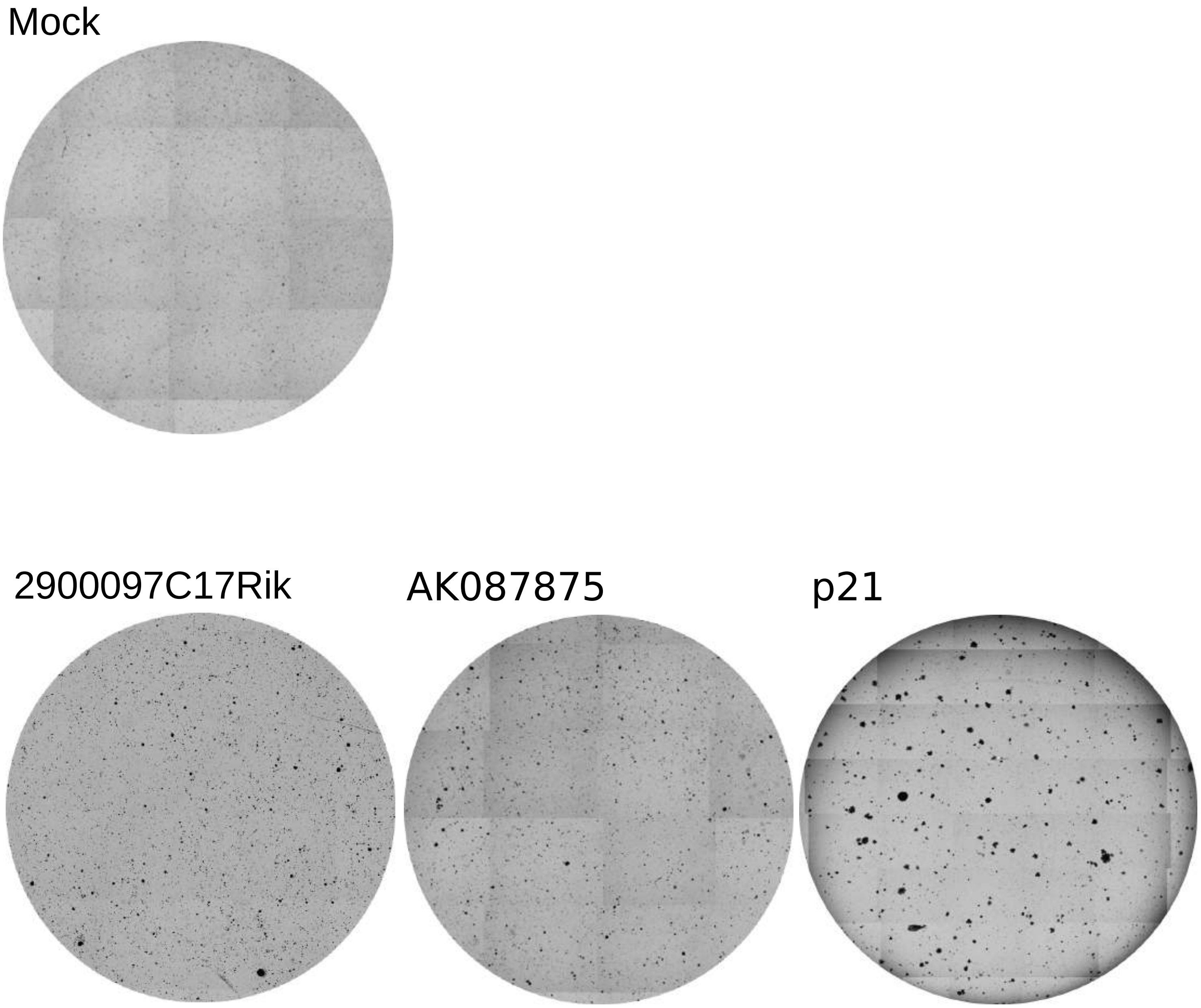
Mock
2900097C17Rik
AK087875
p21

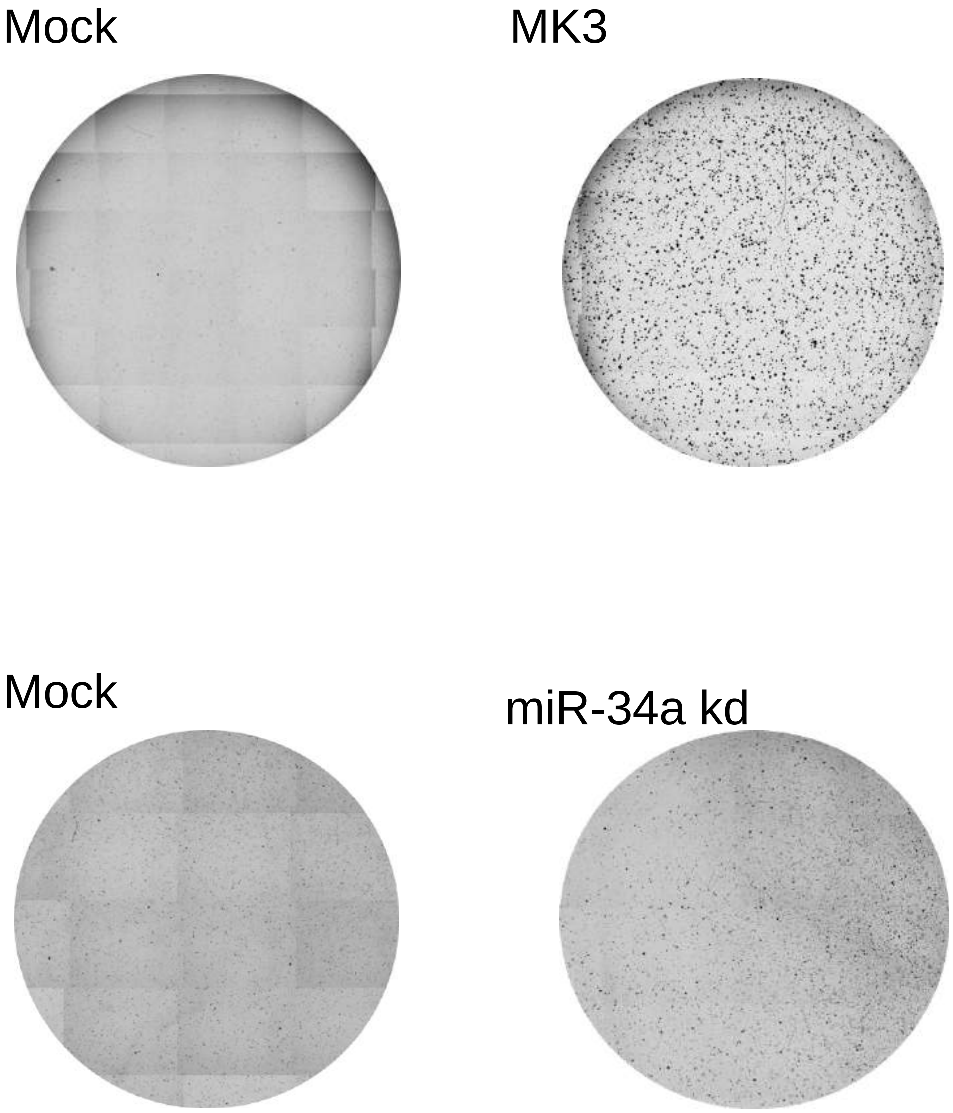
Mock
MK3
Mock
miR-34a kd

D. Colony forming assays using miR-34a expressing and knockdown cells confirm the growth-suppressive role of the microRNA

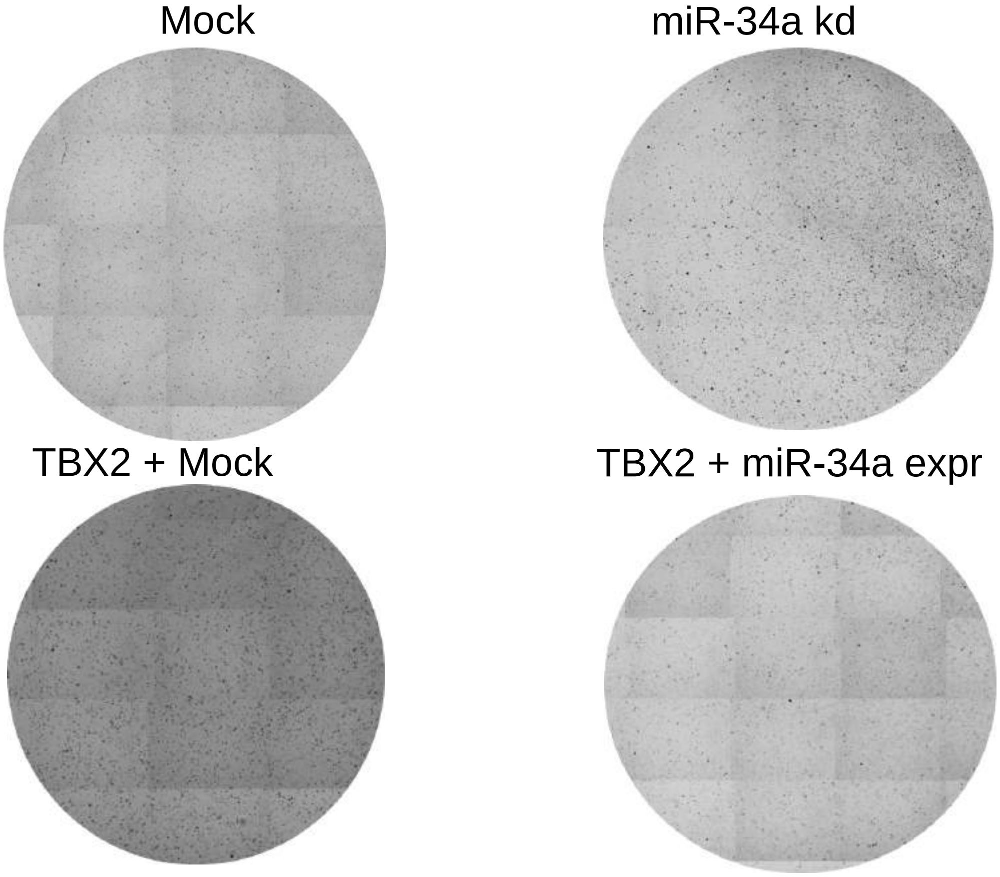

E. Colony forming assays using MK3 expressing and knockdown cells confirm the growth-suppressive role of thep38 effector kinase

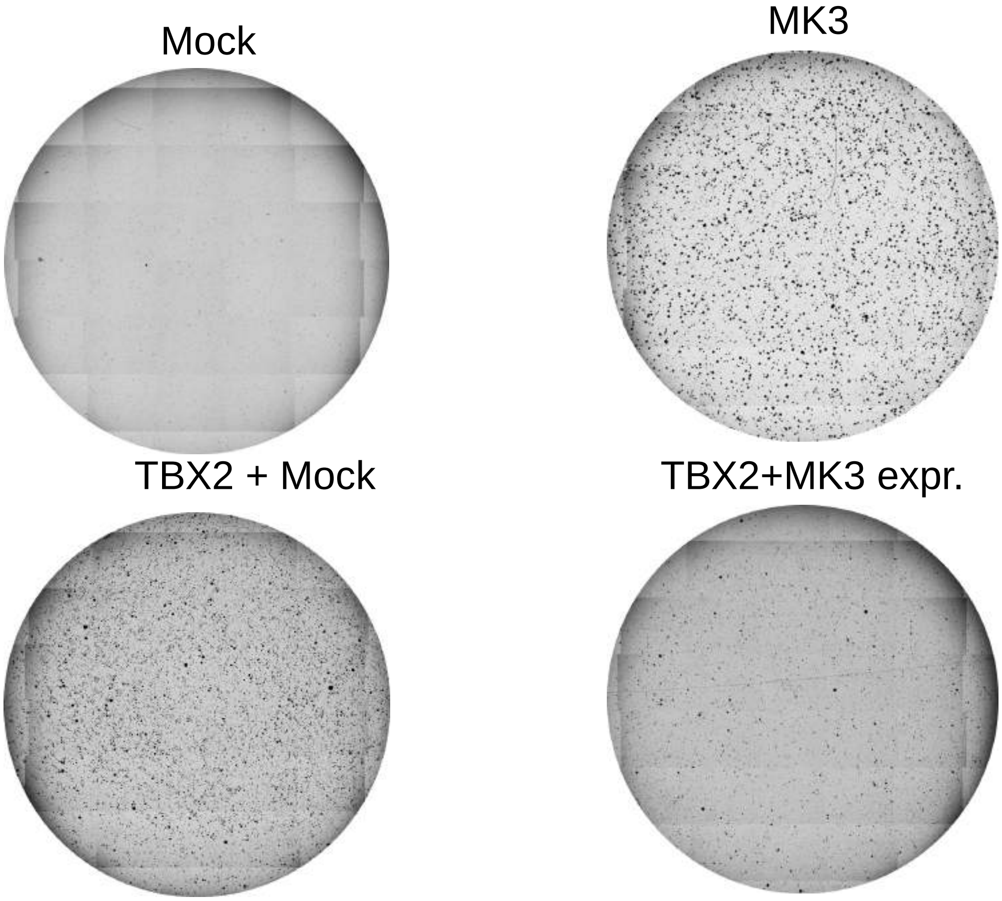

F. Ingenuity network analysis based on 23 hits that were repressed (1.66-11.9 fold) in both transformed genotypes during anchorless proliferation.

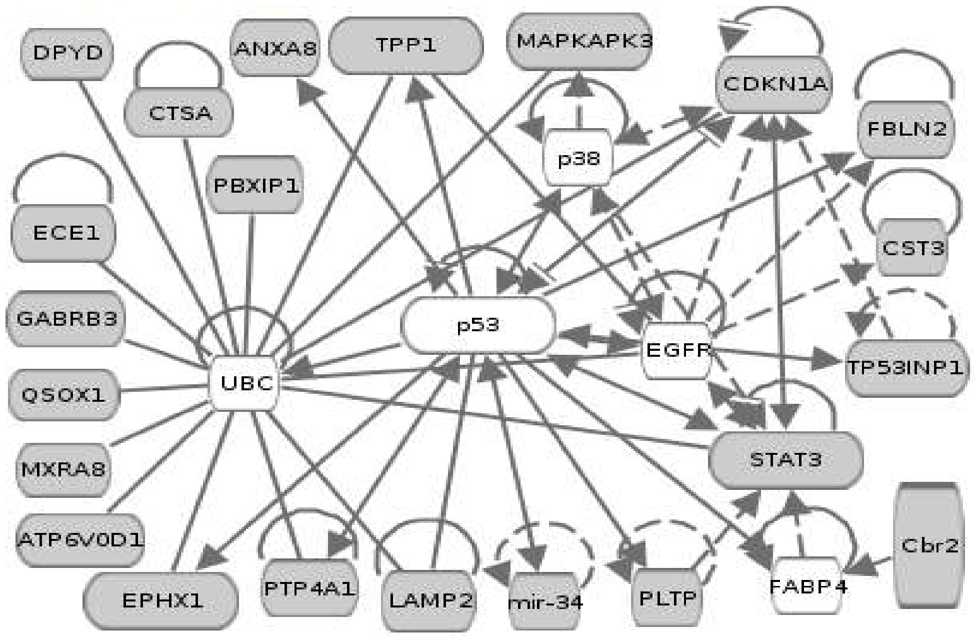

© 2000-2015 QIAGEN. All rights reserved.

G. Colony forming assays of cells expressing knockdown vector targeting Tug1, GabrA5, B3, G3 and ADO. Addition of taurin rescues the growth arrest of ADO knockdown cells.

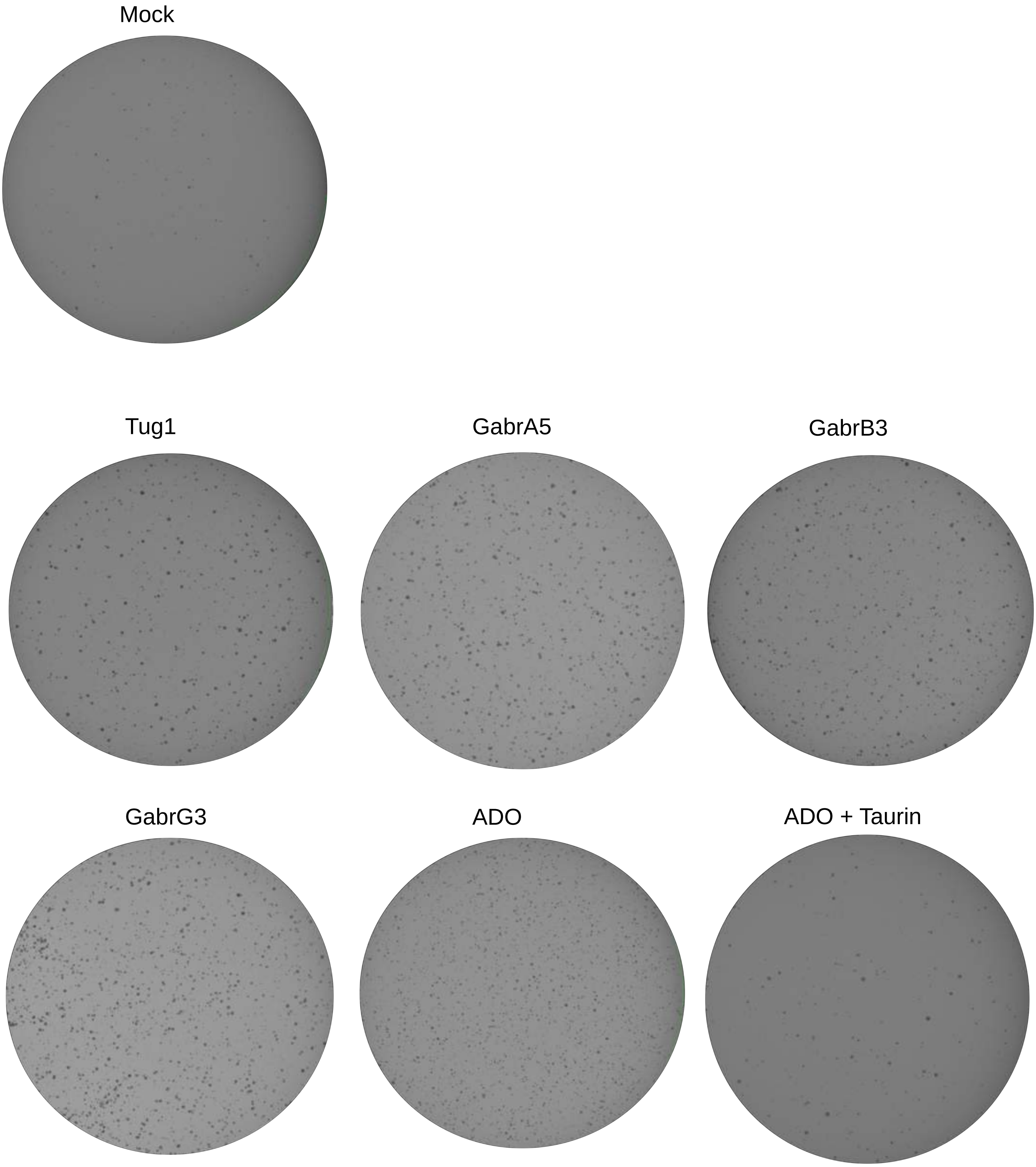

Supplement: S2 Fig — A. Newly designed shRNA sequences inserted into pRETRO Super vectors used for knockdown of identified suppressor transcripts. B. Level of knockdown achieved for a subset of these shRNA vectors in typical experiments as measured by quantitative PCR. C. Retesting of selected hits. Newly designed shRNA vectors targeting 17 selected suppressor transcripts were tested for their effect on proliferation in colony forming assays in methylcellulose. D. miR-34a is a suppressor of anchorless proliferation. Colony forming assays showing increased proliferation of Rb-/-107-/- RASV12 MEFs in methylcellulose upon knockdown (kd) of the non-coding suppressor transcript miR-34a. Viral co-expression (expr) of miR-34a in Rb-/-107-/- RASV12 TBX2 MEFs largely reverted transformation. E. The p38 stress pathway suppresses anchorless proliferation. Colony forming assays showing increased proliferation of Rb-/-107-/- RASV12 MEFs in methylcellulose upon knockdown (kd) of MK3, Map2k6 and Map2k3. Viral co-expression (expr) of MK3 in Rb-/-107-/- RASV12 TBX2 MEFs largely reverted transformation. In C, D and F, pictures of wells were taken 3 weeks after 5x10^4^ cells were seeded per well. The Luc vector (Mock) was used as a negative control for miR-34a. F. Network analysis of hits. Network revealed by Ingenuity analysis using 23 hits that were induced (1.66–7.05 fold) in non-transformed DKO RASV12 cells after loss of anchoring, but repressed (1.66–11.9 fold) in both transformed genotypes during anchorless proliferation. (PDF) [file pone.0196979.s004.pdf]
