# Supplementary material for: RNAi screening of subtracted transcriptomes reveals tumor suppression by taurine-activated GABAA receptors involved in volume regulation
Source: PLoS One. 2018 May 22;13(5):e0196979. doi: 10.1371/journal.pone.0196979 (PMC5963783; doi:10.1371/journal.pone.0196979)

Supplementary Figure 3. Induction of suppressor proteins upon loss of anchoring. A. Western blots showing strong and weak p21 protein induction in anchor deprived DKO RAS^{V12} and DKO RAS^{V12} TBX2 cells, respectively. Lanes derived from different sections of a single gel are shown. **B.** GabrA5 and GabrB3 protein levels in attached and non-attached DKO RAS^{V12} and DKO RAS^{V12} p53kd cells. Lanes shown represent different sections of the same gel.

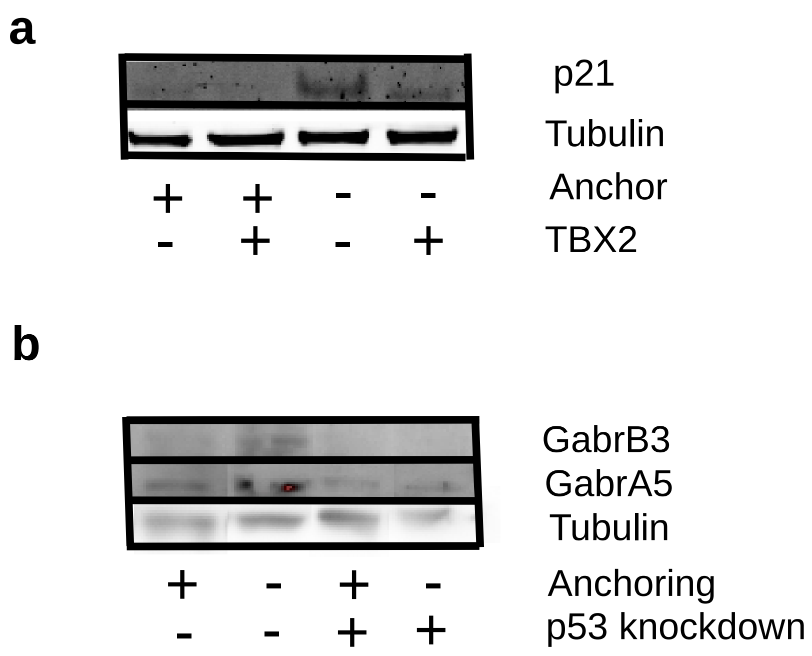

Supplement: S3 Fig — A. Western blots showing strong and weak p21 protein induction in anchor deprived DKO RASV12 and DKO RASV12 TBX2 cells, respectively. Lanes derived from different sections of a single gel are shown. B. GabrA5 and GabrB3 protein levels in attached and non-attached DKO RASV12 and DKO RASV12 p53kd cells. Lanes shown represent different sections of the same gel. (PDF) [file pone.0196979.s005.pdf]
