# Supplementary material for: RNAi screening of subtracted transcriptomes reveals tumor suppression by taurine-activated GABAA receptors involved in volume regulation
Source: PLoS One. 2018 May 22;13(5):e0196979. doi: 10.1371/journal.pone.0196979 (PMC5963783; doi:10.1371/journal.pone.0196979)

Supplementary Figure 4. Expression of $GABA_A$ receptor subunits. RNA sequencing revealed low expression of particular $GABA_A$ receptor subunits in attached DKO RAS^{V12} (blue), in anchorless DKO RAS^{V12} (red) and in anchorless DKO RAS^{V12} p53kd cells (yellow). Subunits GabrB3 and GabrA5 were upregulated by loss of anchoring and repressed by p53. For comparison, their expression was over 325-fold lower than that of Hprt.

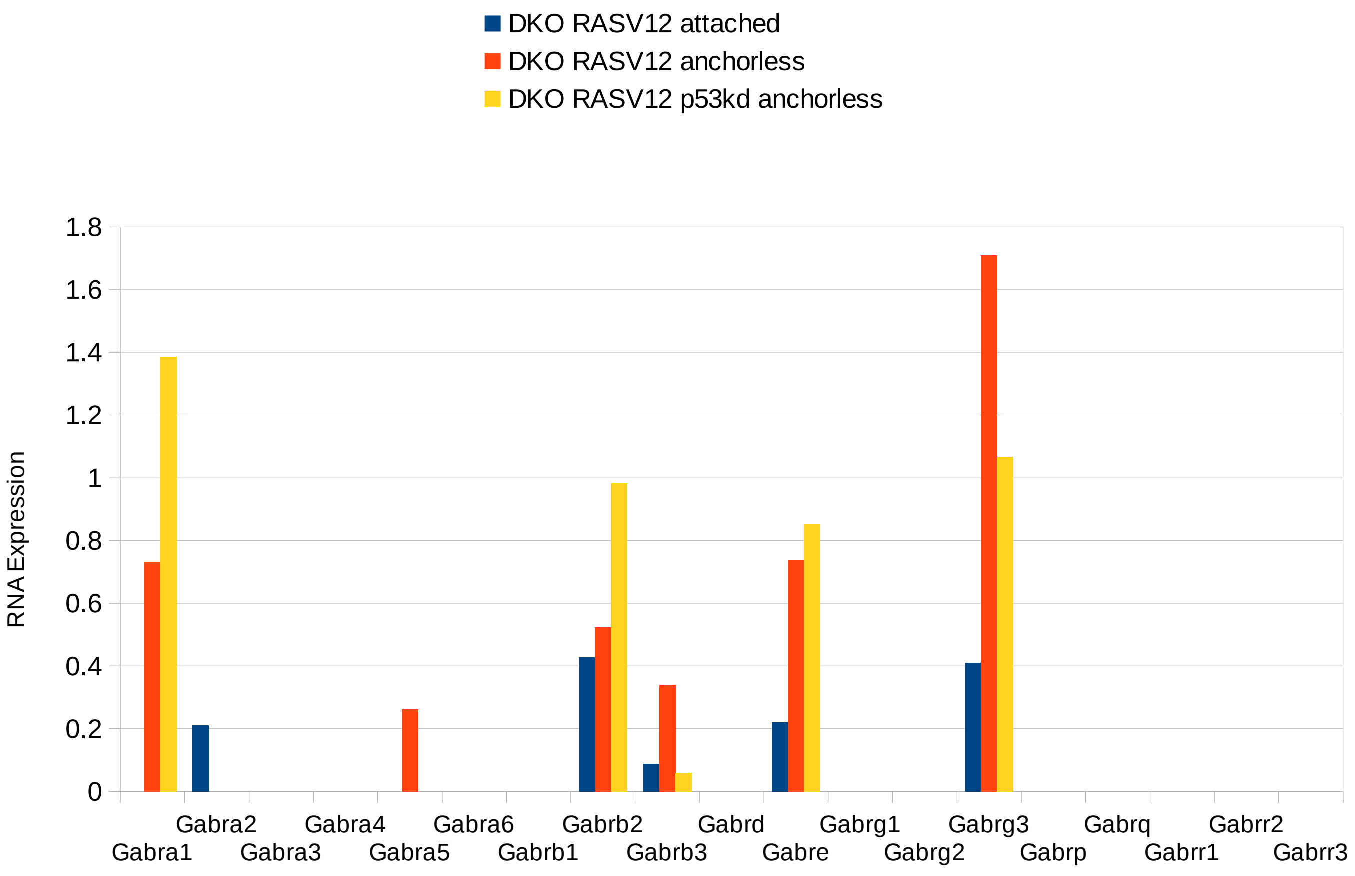

Supplement: S4 Fig — RNA sequencing revealed low expression of particular GABAA receptor subunits in attached DKO RASV12 (blue), in anchorless DKO RASV12 (red) and in anchorless DKO RASV12 p53kd cells (yellow). Subunits GabrB3 and GabrA5 were regulated both by loss of anchoring and by p53. For comparison, their expression was over 325-fold lower than that of Hprt. (PDF) [file pone.0196979.s006.pdf]
