# Supplementary material for: RNAi screening of subtracted transcriptomes reveals tumor suppression by taurine-activated GABAA receptors involved in volume regulation
Source: PLoS One. 2018 May 22;13(5):e0196979. doi: 10.1371/journal.pone.0196979 (PMC5963783; doi:10.1371/journal.pone.0196979)

Supplementary Figure 5. GABAA receptor subunit expression in cholangiocarcinomas.
A. mRNA expression of GABRA5 and Ado did not change significantly in 104 cholangiocarcinomas (red; T) compared to normal bile ducts (white; N) and surrounding liver tissue (green; SL). **B.** Survival of 104 patients is not affected by the expression of GABRB3 in cholangiocarcinomas. Cholangiocarcinomas were classified as “higher than mean RNA expression” (black line) or “lower than mean RNA expression” (red line) and Kaplan Meyer curves were generated for both groups. **C.** Scatterplot showing strong negative correlation between GABRA5 gene expression and Ki67 gene expression in 37 cholangiocarcinomas present in the TCGA database (slope = -1,694667415; r.sq = 0,2608221533; p value = 0,0015).

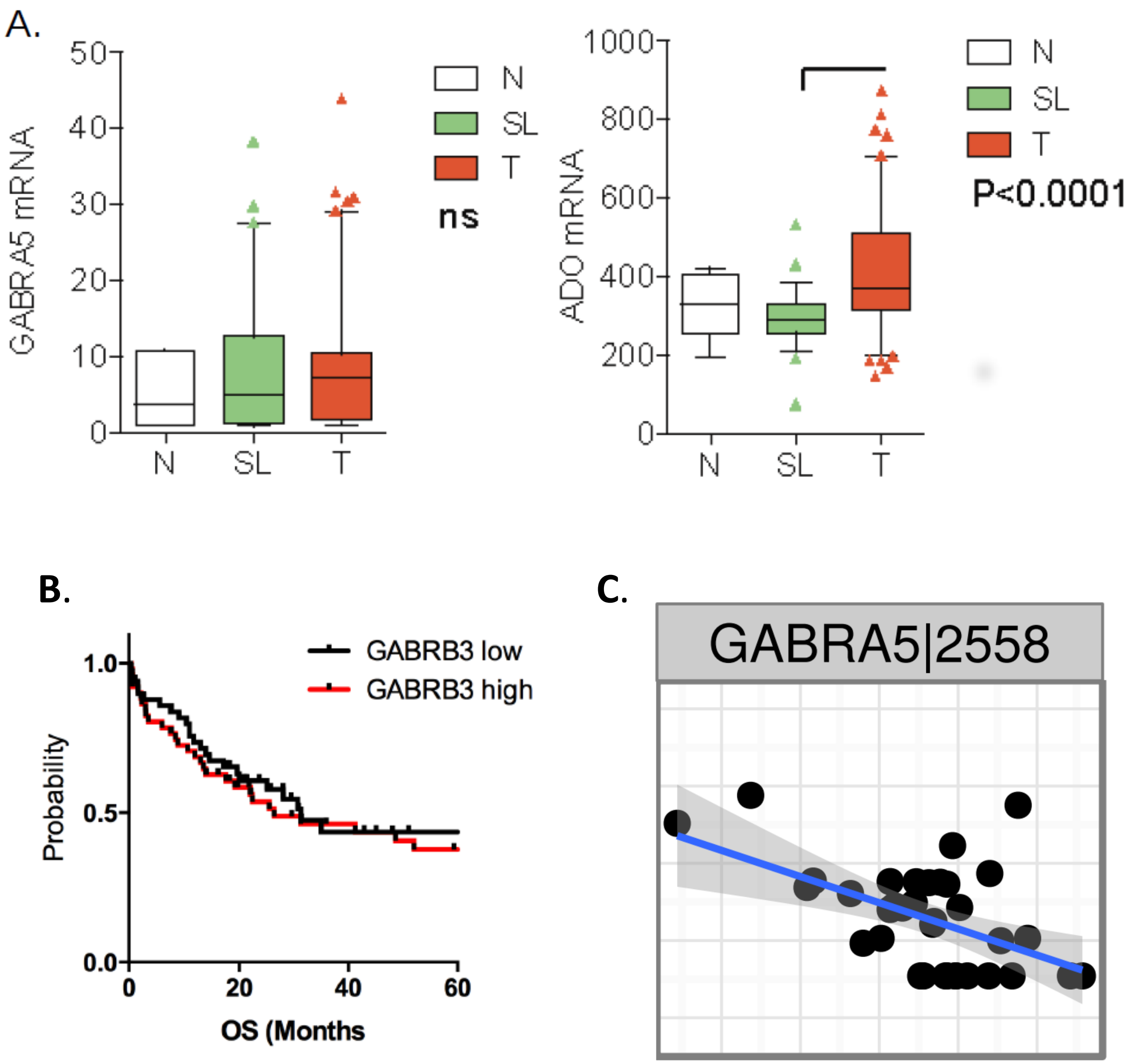

Supplement: S5 Fig — A. mRNA expression of GABRA5 and Ado did not change in 104 cholangiocarcinomas (red; T) compared to normal bile ducts (white; N) and surrounding liver tissue (green; SL) B. Survival of 104 patients is not affected by the expression of GABRB3 in cholangiocarcinomas. Cholangiocarcinomas were classified as “higher than mean RNA expression” (black line) or “lower than mean RNA expression” (red line) and Kaplan Meyer curves were generated for both groups. C. Scatterplot showing strong negative correlation between GABRA5 gene expression and Ki67 gene expression in 37 cholangiocarcinomas present in the TCGA database (slope = -1,694667415; r.sq = 0,2608221533; p value = 0,0015). (PDF) [file pone.0196979.s007.pdf]
